# Supplementary material for: Single cell transcriptomics shows that malaria promotes unique regulatory responses across multiple immune cell subsets
Source: Nat Commun. 2023 Nov 15;14:7387. doi: 10.1038/s41467-023-43181-7 (PMC10651914; doi:10.1038/s41467-023-43181-7)
Supplement: Supplementary file 1 — Supplementary Information [file 41467_2023_43181_MOESM1_ESM.pdf]

Supplementary Materials

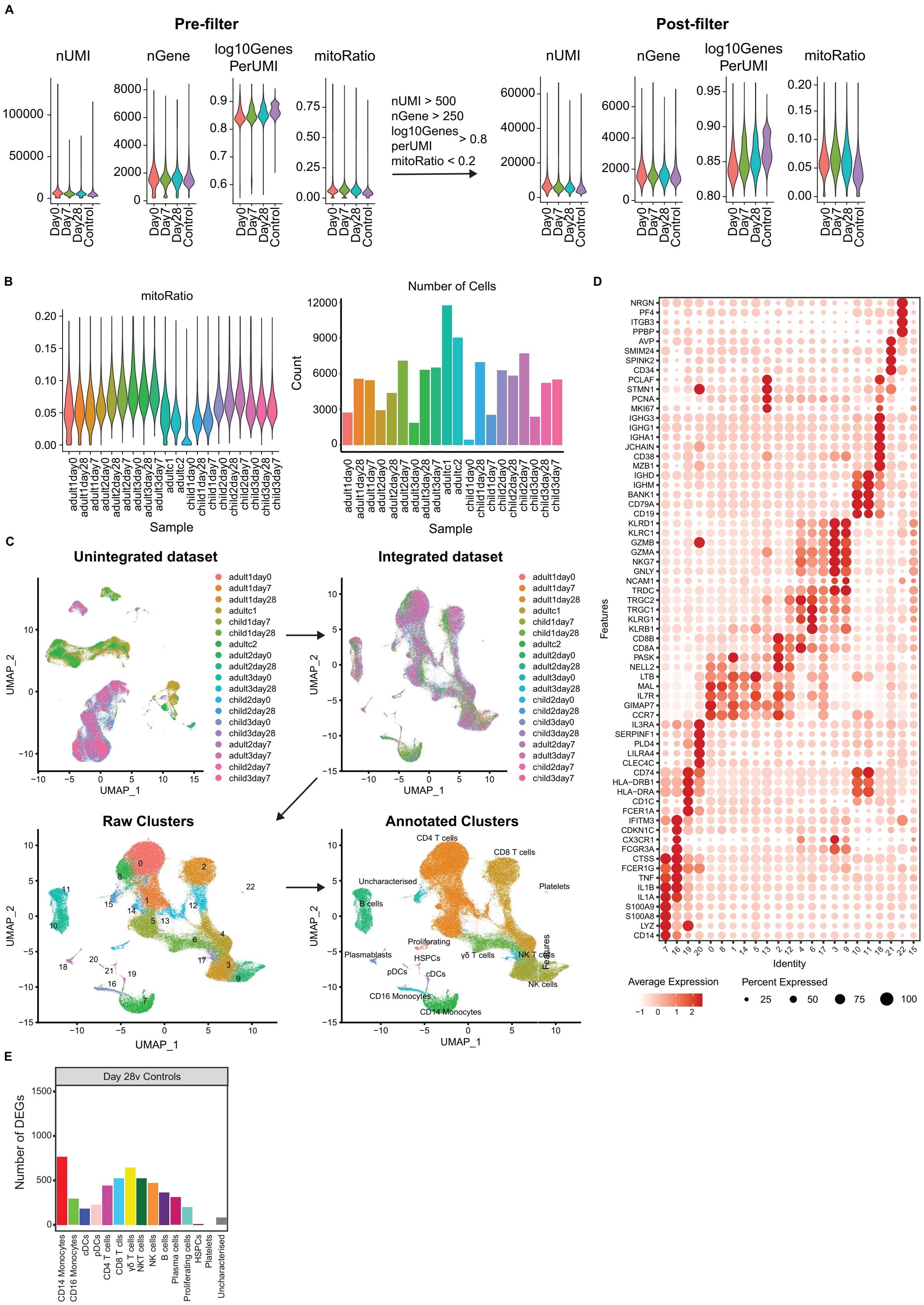

Supplementary Fig. 1: scRNAseq pre-processing QC. (**A**) Pre- and post-filter violin plots of nUMI

(transcript counts per cell), nGene (gene counts per cell), log10GenesPerUMI, mitoRatio (percentage of mitochondrial genes per cell). Cells removed based on filters nUMI > 500, nGene >250, log10GenesPerUMI >0.8, mitoRatio < 0.2. (**B**) Violin plot of mitoRatio bar plot of cell counts from each sample to justify removal of sample "child1day0". (**C**) Workflow of integration and annotation of Seurat cluster by UMAP visualisation. UMAPs of coloured by sample pre- and post-integration by sample. Integrated UMAP of cluster distribution and numbering based on Seurat default shared nearest neighbour (SNN) clustering algorithm at a resolution of 0.6. Annotated cluster UMAP to define cell subset, based on (**D)** expression of canonical genes in dot plot (See also Fig. 1B). (**E**) Number of DEGs per annotated cell subset comparing malaria patients 28 days post-treatment and endemic healthy controls. Analysis performed by and graphs generated with Seurat v4.

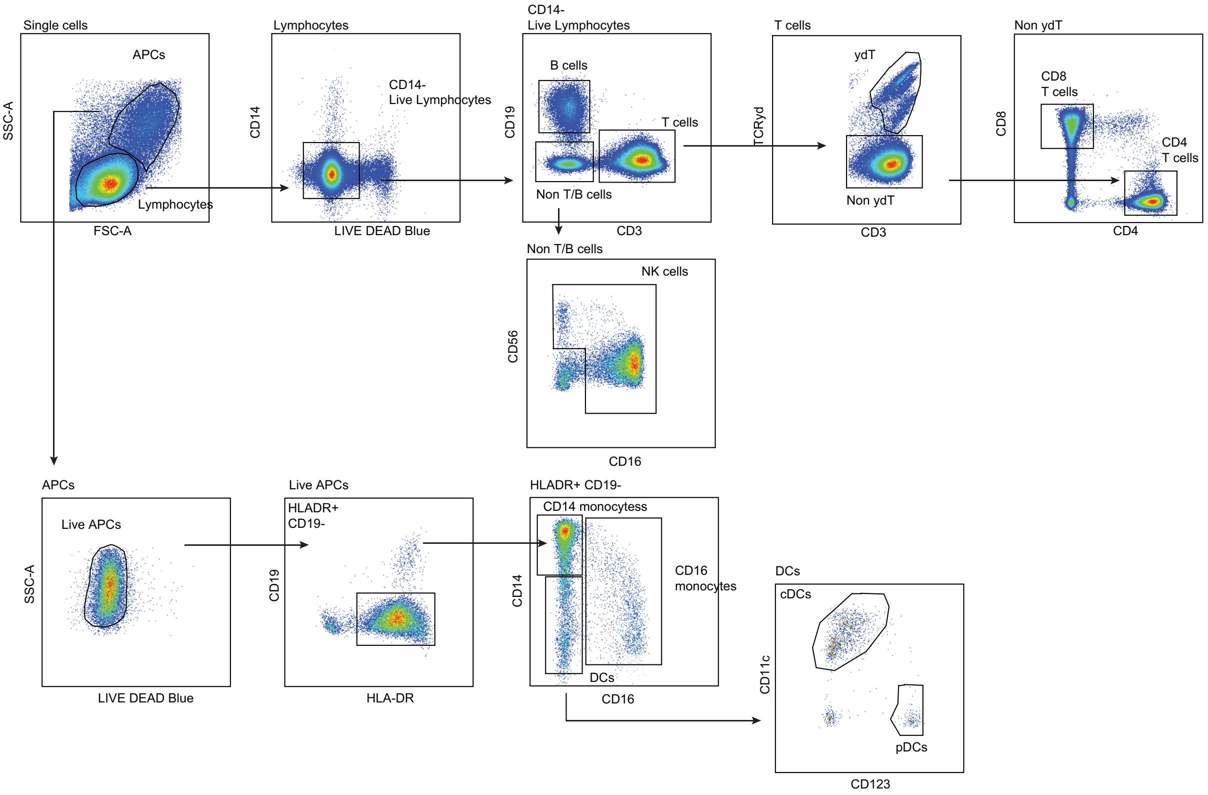

Supplementary Fig. 2: Gating strategy of Flow cytometric cell phenotyping comparison of scRNAseq samples. Peripheral blood mononuclear (PBMCs) from each individual analysed for scRNAseq were analysed by flow cytometry to identify major cell subsets. Gating strategy is outlined.

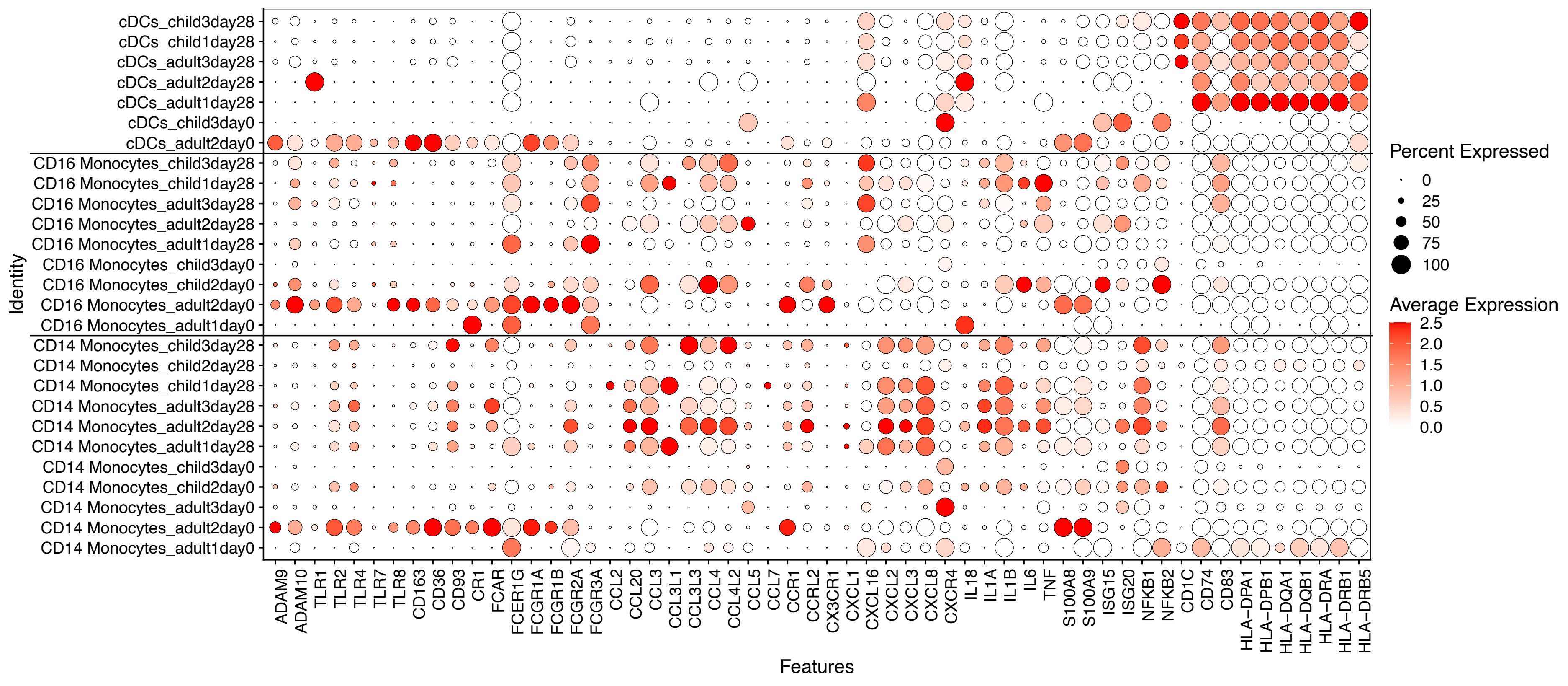

Supplementary Fig. 3: Myeloid cell malaria driven differential expressed genes as average expression of each donor at day 0 and day 28. Average expression of DEGs identified during malaria in monocytes and cDCs shown as average expression on individual level at day 0 and day 28 timepoints.

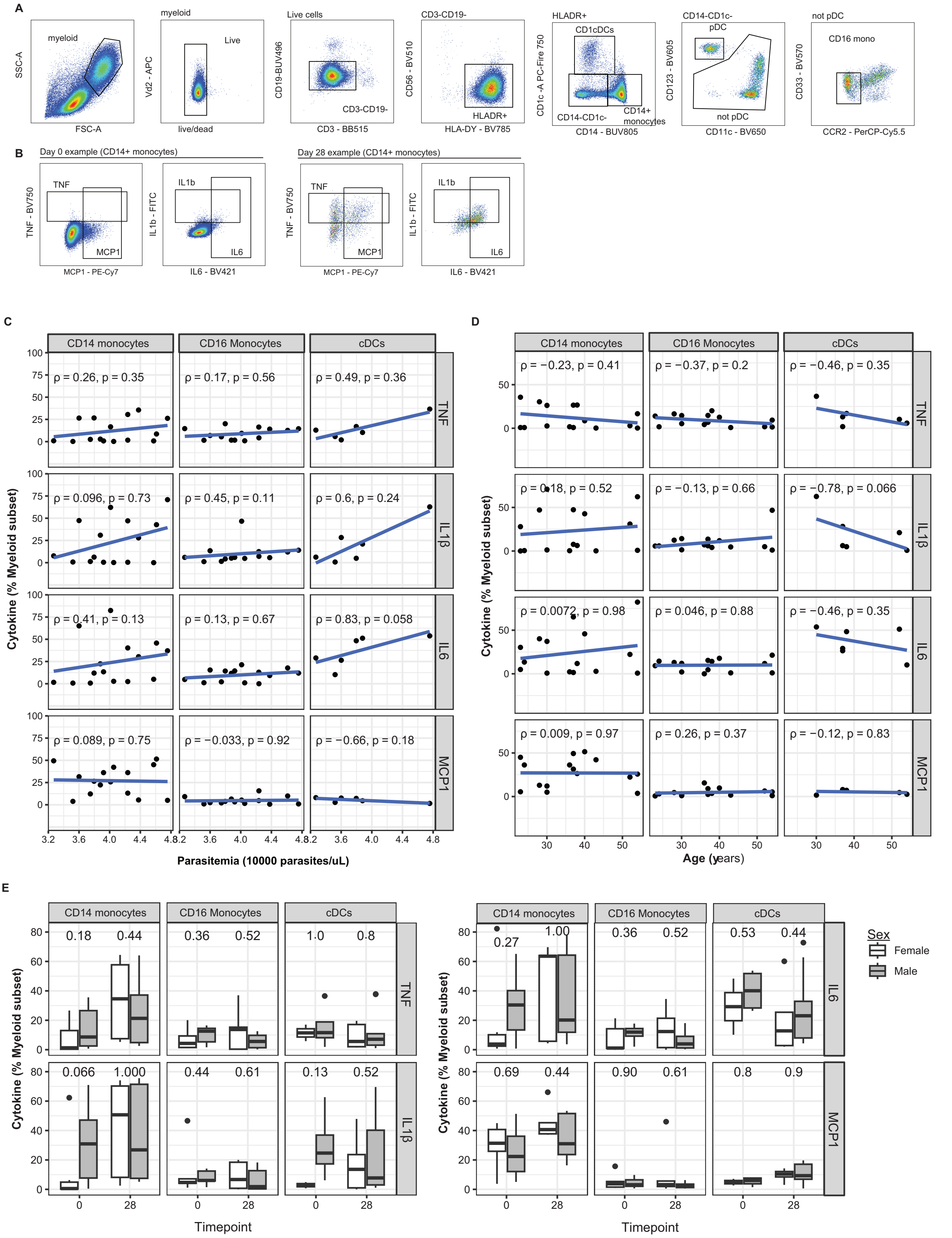

Supplementary Fig. 4: Myeloid cell subset *ex vivo* cytokine production analysis. To investigate key changes to myeloid cell cytokine production, PBMCs from patients with malaria (day 0) and 28 days post treatment were rested *ex vivo* for 4 hours and cytokine captured by addition of Monensin then

measured by flow cytometry. (**A**) Gating strategy to identify $CD14^+$ monocytes, $CD16^+$ monocytes and $CD1c^+$ DCs. Due to the down regulation of CD16 in culture cells, alternative gating strategy as HLA-DR^+/$CD1c^-$/$CD14^-$/$CD123^-$/$CCR2^-$/$CD33^-$ was used. (**B**) Representative FACS gating of TNF, MCP1, IL6, IL1β expression from CD14 monocytes at day 0 and day 28. Frequency of cytokine positive $CD14^+$ monocytes, $CD16^+$ monocytes and $CD1c^+$ DCs linear association with (**C**) *Plasmodium falciparum* parasitemia (10000 parasites/μL) and (**D**) age (years). Scatterplot line performed by linear regression, Spearman's rank correlation. (**E**) Frequency of cytokine positive $CD14^+$ monocytes, $CD16^+$ monocytes and $CD1c^+$ DCs at day 0 and day 28 compared between sexes. Box plots show the median and IQR of volunteers, group comparisons performed by Mann Whitney U test.

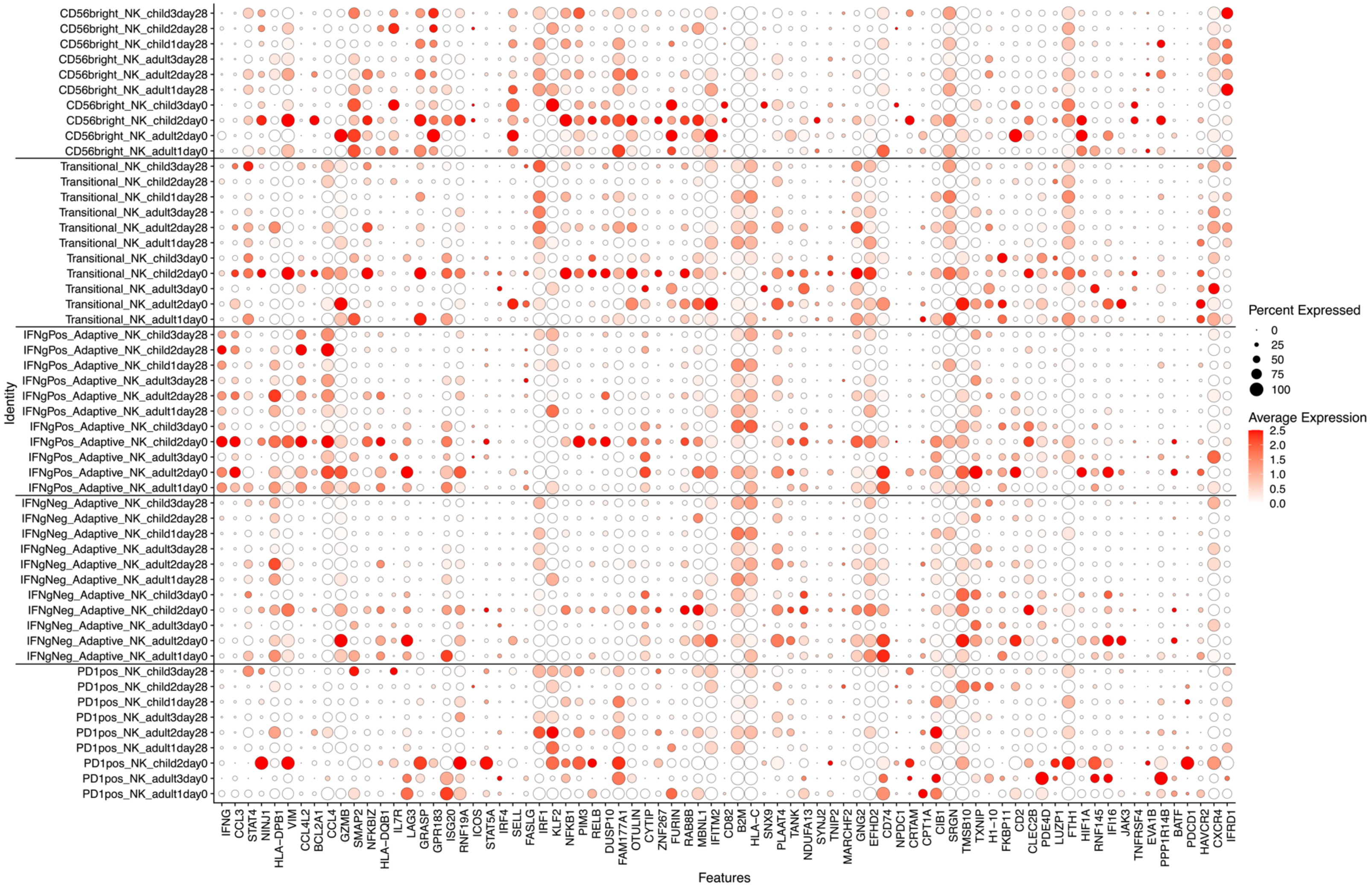

Supplementary Fig. 5: Malaria driven differential expressed genes in NK cell subsets as average expression of each donor at day 0 and day 28. Average expression level (white to red) and percentage of expression (dot size) in cell population of genes in the individual at day 0 and day 28 from each cell cluster.

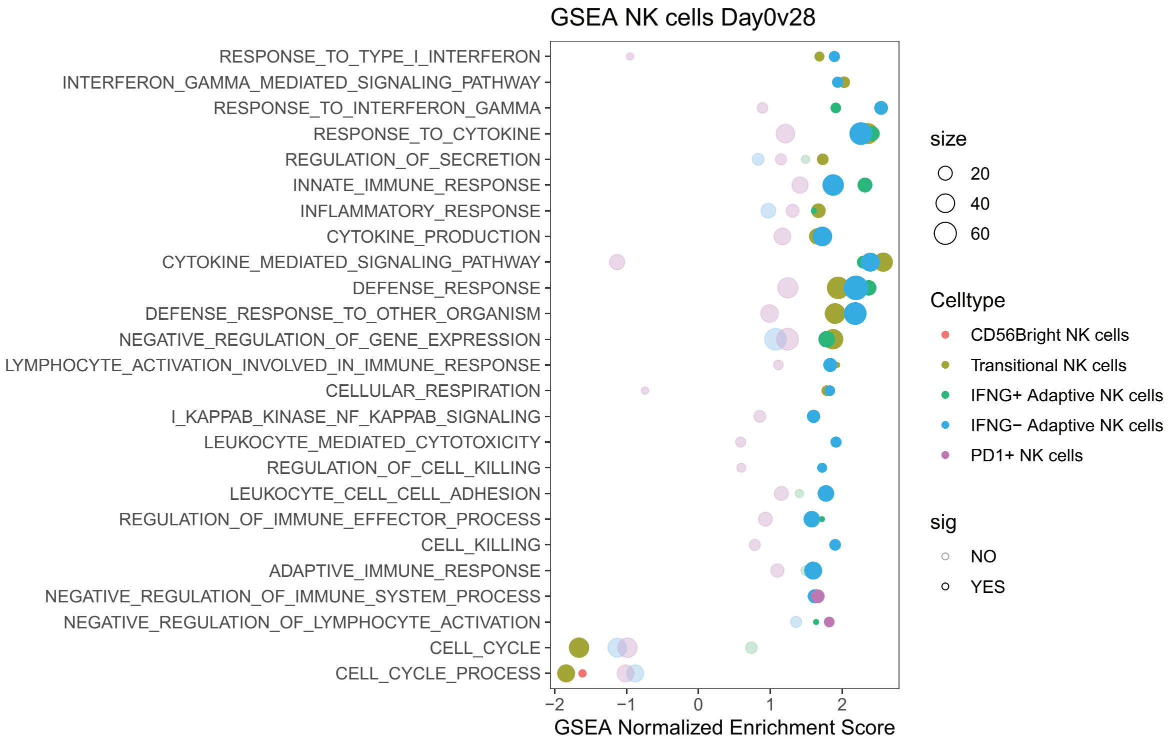

Supplementary Fig. 6: GSEA of DEGs from NK cell subsets. Differentially expressed genes (DEGs) identified between day 0 (*n*=5) and day 28 (*n*=6) for each NK cell subset were analysed using GSEA publicly available software (https://www.gsea-msigdb.org/gsea/index.jsp). Enriched signatures for each subset indicated.

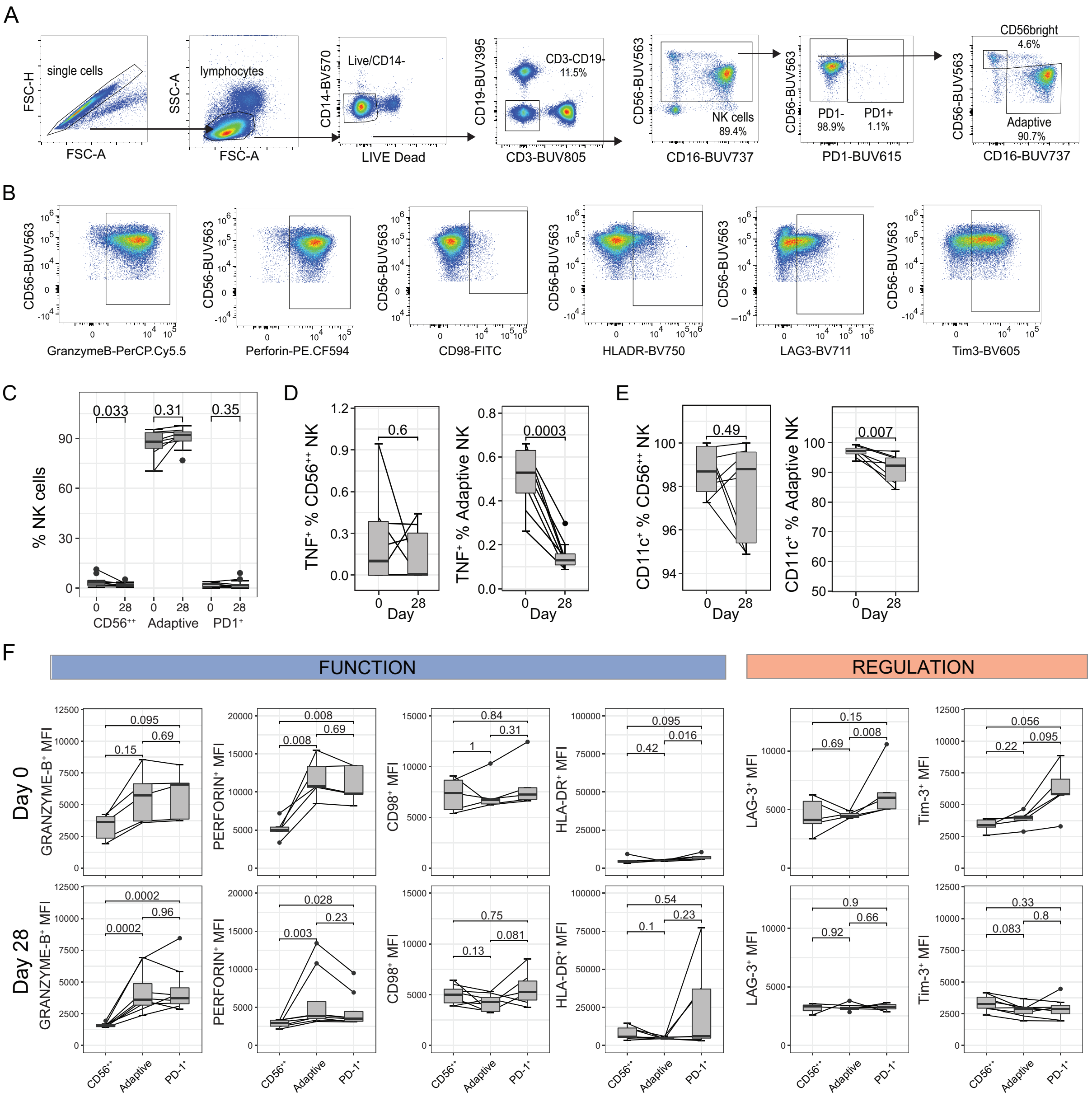

Supplementary Fig. 7: Flow cytometric analysis of NK cells. PBMCs from individuals with acute *Plasmodium falciparum* infection (day 0, *n*=11) and 28-days post-infection (day 28, *n*=17), were analysed *ex vivo* to identify NK cell protein expression of DEGs. (**A**) Gating strategy to identify PD1$^+$, CD56 bright (CD56^{++}) and Adaptive NK subsets. (**B**) Representative FACS gating of Granzyme-B, Perforin, CD98, ICOS, HLA-DR, LAG-3 and TIM-3 against CD56 expression at day 0 in total NK population. (**C**) Proportion of NK cell subsets at day 0 (*n*=11) and day 28 (*n*=17). Expression of (**D**) TNF and **E**) CD11c expression by CD56^{++} and Adaptive NK cells at day 0 (*n*=8) and day 28 (*n*=8). (**F**) Mean fluorescence intensity (MFI) of Granzyme-B$^+$, Perforin$^+$, CD98$^+$, HLA-DR$^+$, LAG-3$^+$ or Tim-3$^+$ events in CD56^{++}, Adaptive and PD1$^+$ NK cell populations at day 0 and day 28 (*n*=5 day 0, *n*=8 day 28). **G)** For all data, box plots show the median and IQR of volunteers, lines represent paired

observations, group comparisons performed by Mann Whitney U test and paired group comparisons performed by Wilcoxon signed rank test.

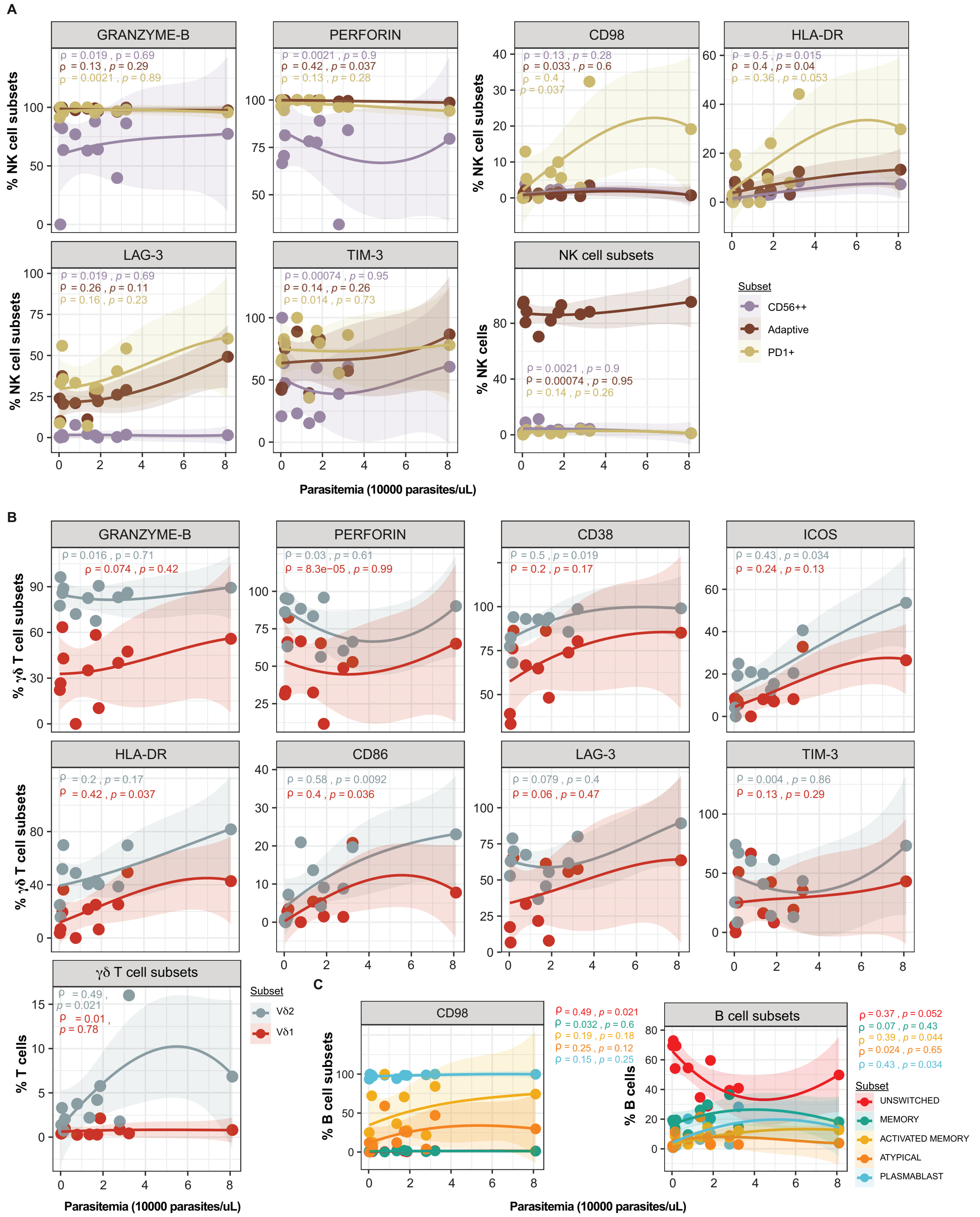

Supplementary Fig. 8: Association of blood parasitemia with lymphocyte *ex vivo* phenotypes and frequencies. PBMCs from individuals with acute *Plasmodium falciparum* infection (day 0, *n*=11) were analysed *ex vivo*. Cell frequencies and marker expression by (**A**) NK, (**B**) γδ T and (**C**) B cells associated with *Plasmodium falciparum* parasitemia (10000 parasites/μL). Scatterplot line performed by Loess regression, Spearman's rank correlation.

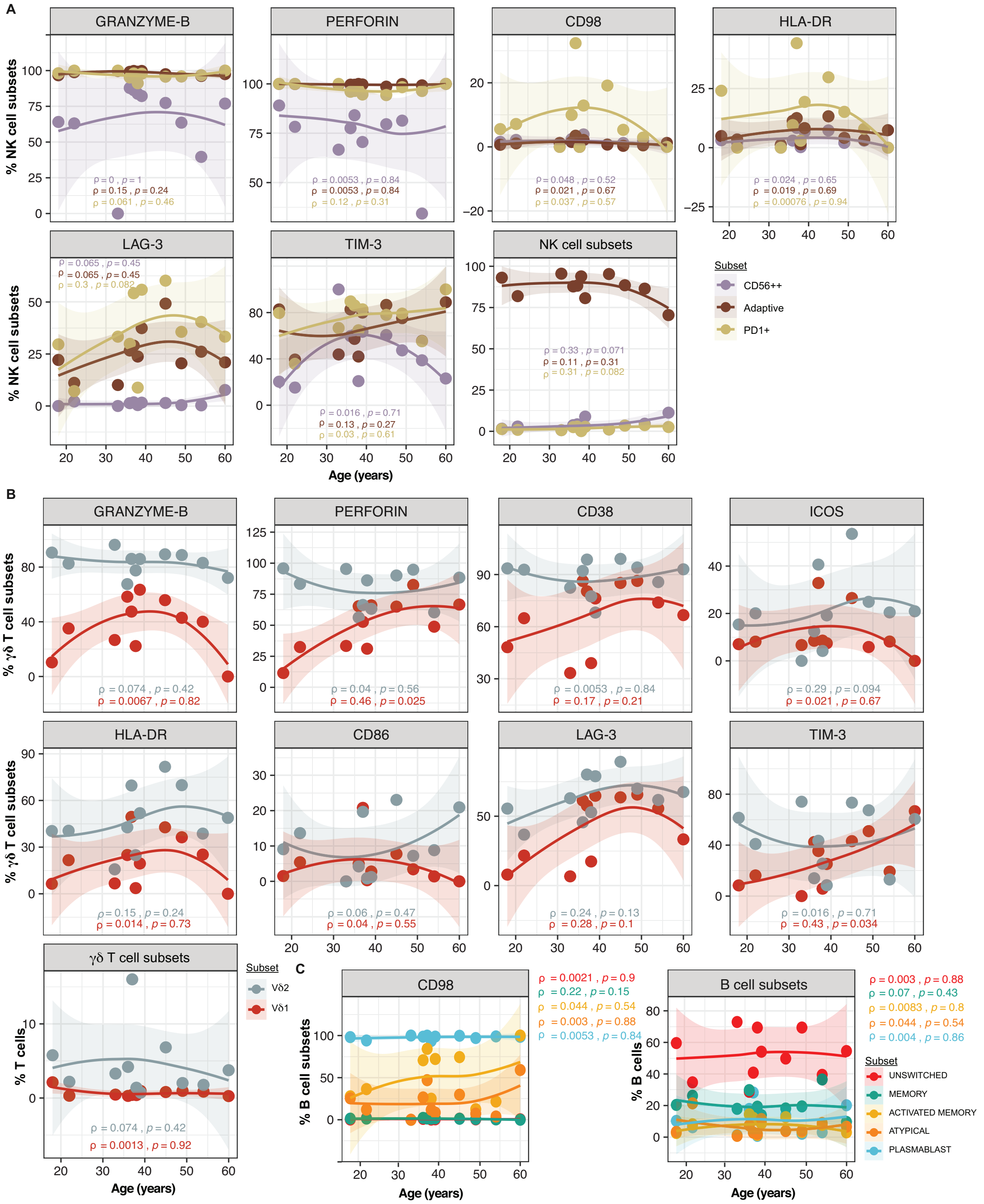

Supplementary Fig. 9: Association of age with lymphocyte *ex vivo* phenotypes and frequencies. PBMCs from individuals with acute *Plasmodium falciparum* infection (day 0, *n*=11) were analysed *ex vivo*. Cell frequencies and marker expression by (**A**) NK, (**B**) γδ T and (**C**) B cells associated with age (years). Scatterplot line performed by Loess regression, Spearman's rank correlation.

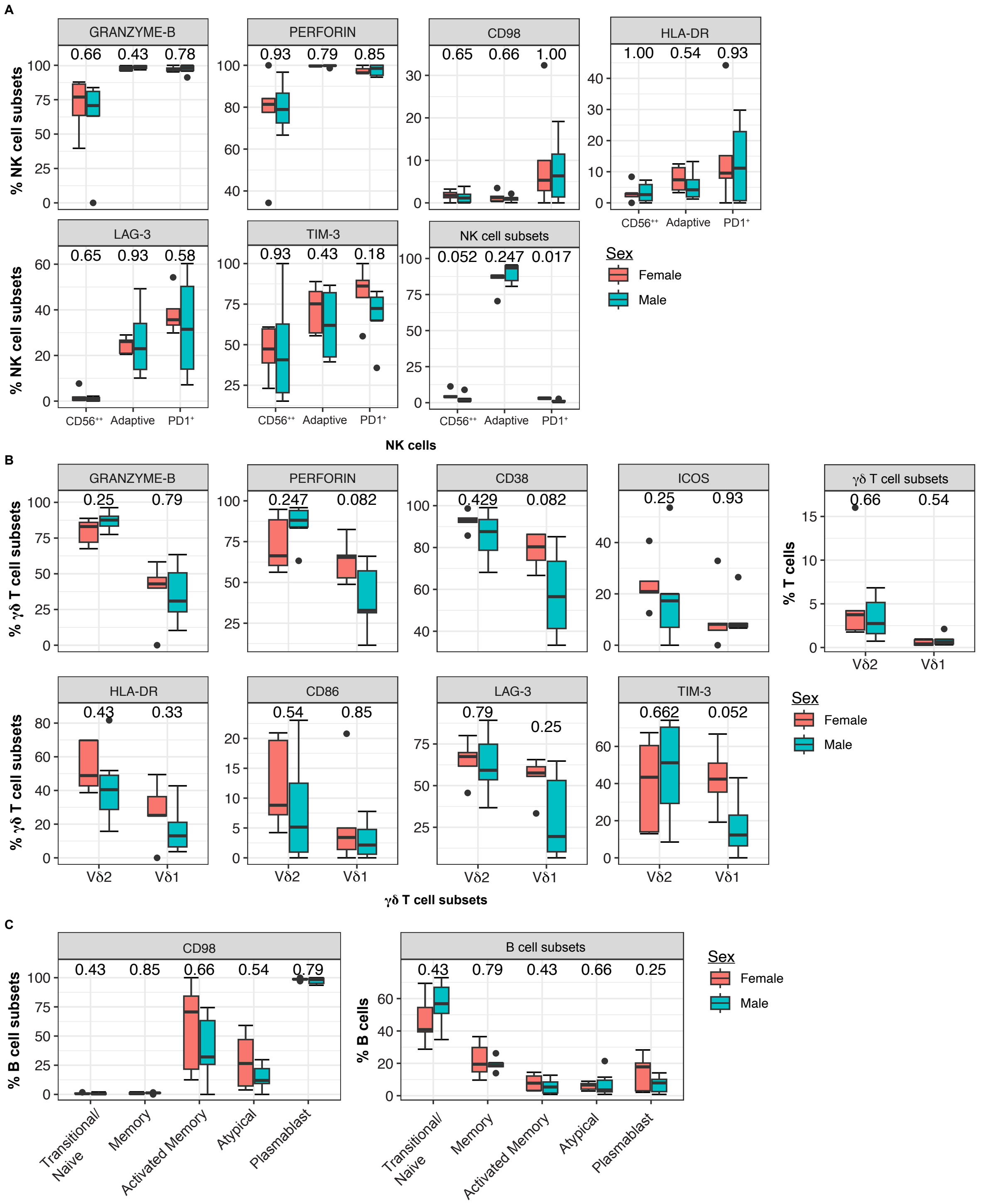

Supplementary Fig. 10: Association of sex with lymphocyte *ex vivo* phenotypes and frequencies. Peripheral blood mononuclear cells from individuals with acute *Plasmodium falciparum* infection (day 0, n=11) were analysed *ex vivo*. Cell frequencies and marker expression by (**A**) NK, (**B**) γδ T and (**C**) B cells associated with sex. Scatterplot line performed by Loess regression, Spearman's rank correlation

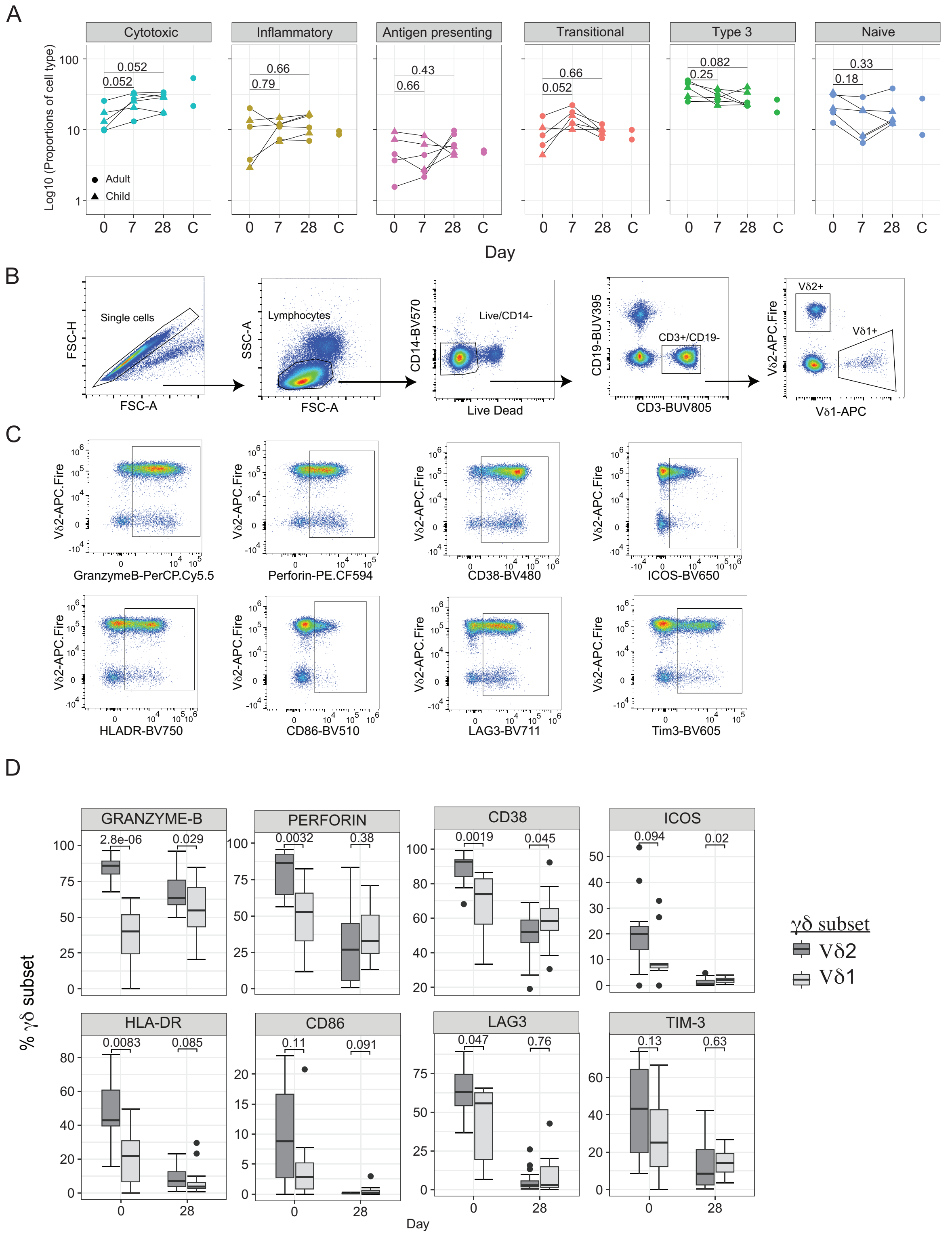

Supplementary Fig. 11: Flow cytometric analysis of γδ T cells. (**A**) Relative proportions of identified of γδ T cell subsets within scRNAseq data during malaria infection (day 0), 7 days and 28 days post treatment, and in healthy uninfected individuals (C) (*n*=5 day 0, *n*=6 day 7, *n*=6 day 28, *n*=2 C). (**B**) FACS gating strategy to identify Vδ2 and Vδ1 γδ T cells. (**C**) Representative FACS gating of Granzyme-B, Perforin, CD38, ICOS, HLA-DR, CD86, LAG-3, TIM-3 on Vδ2 and Vδ1 γδ T cells. (**D**)

Surface marker frequency comparison between Vδ2 and Vδ1 γδ T cells at day 0 (*n*=11) and day 28 (*n*=17) timepoints. For all data, box plots show the median and IQR of volunteers, lines represent paired observations, group comparisons performed by Mann Whitney U test.

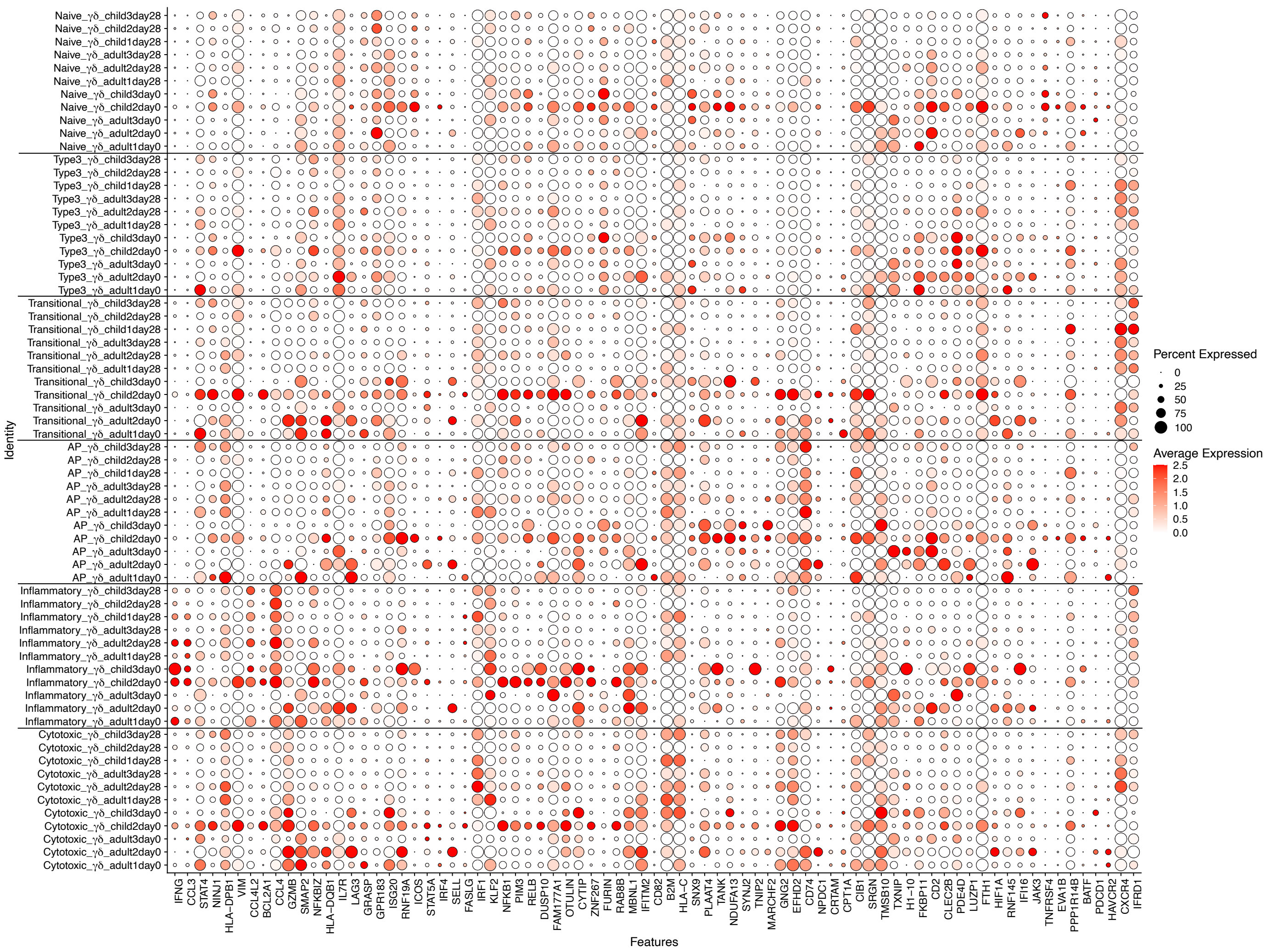

Supplementary Fig. 12: Malaria driven differential expressed genes in γδ T cell subsets as average expression of each donor at day 0 and day 28.

Average expression level (white to red) & percentage of expression (dot size) in cell population in the individual at day 0 and day 28 from each cell cluster.

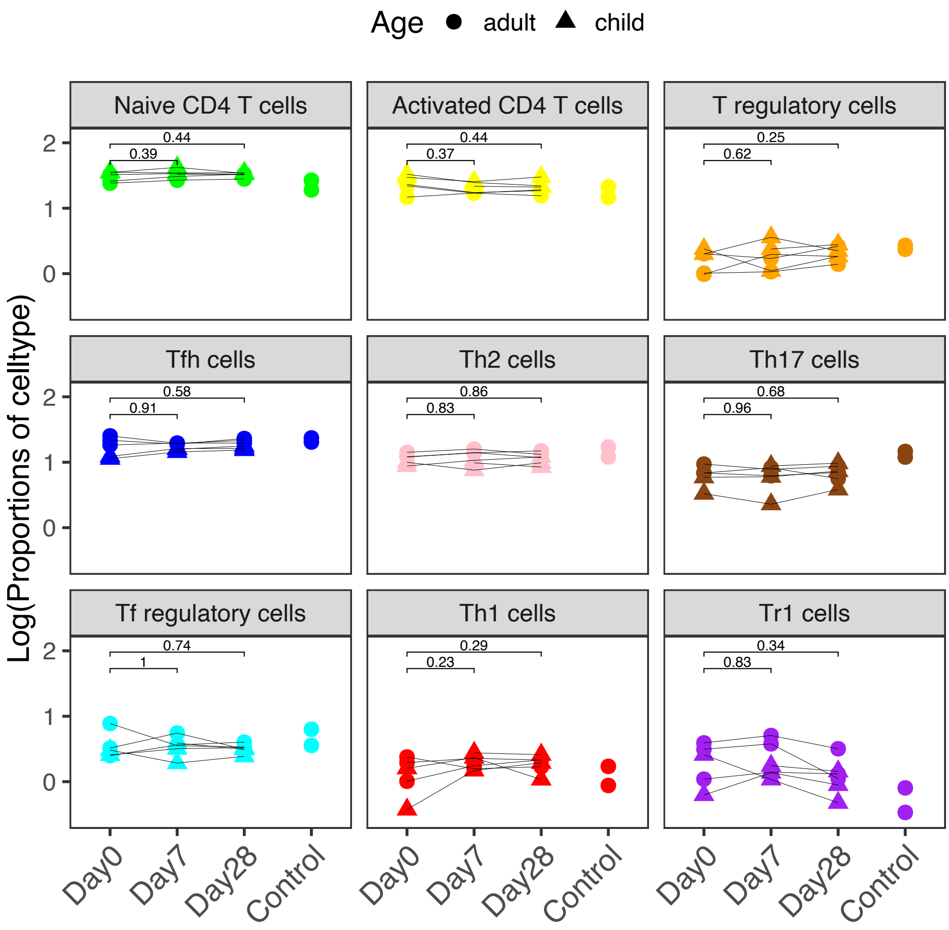

Supplementary Fig. 13: Proportional distribution of CD4 T cell subsets in scRNAseq data. The proportion of each CD4 T cell subset, as a percentage of total CD4 T cells was calculated for each individual and timepoint (*n*=5 day 0, *n*=6 day 7, *n*=6 day 28 and *n*=2 C (healthy uninfected control)). Log10 proportion is shown, group comparisons were performed by Mann Whitney U test.

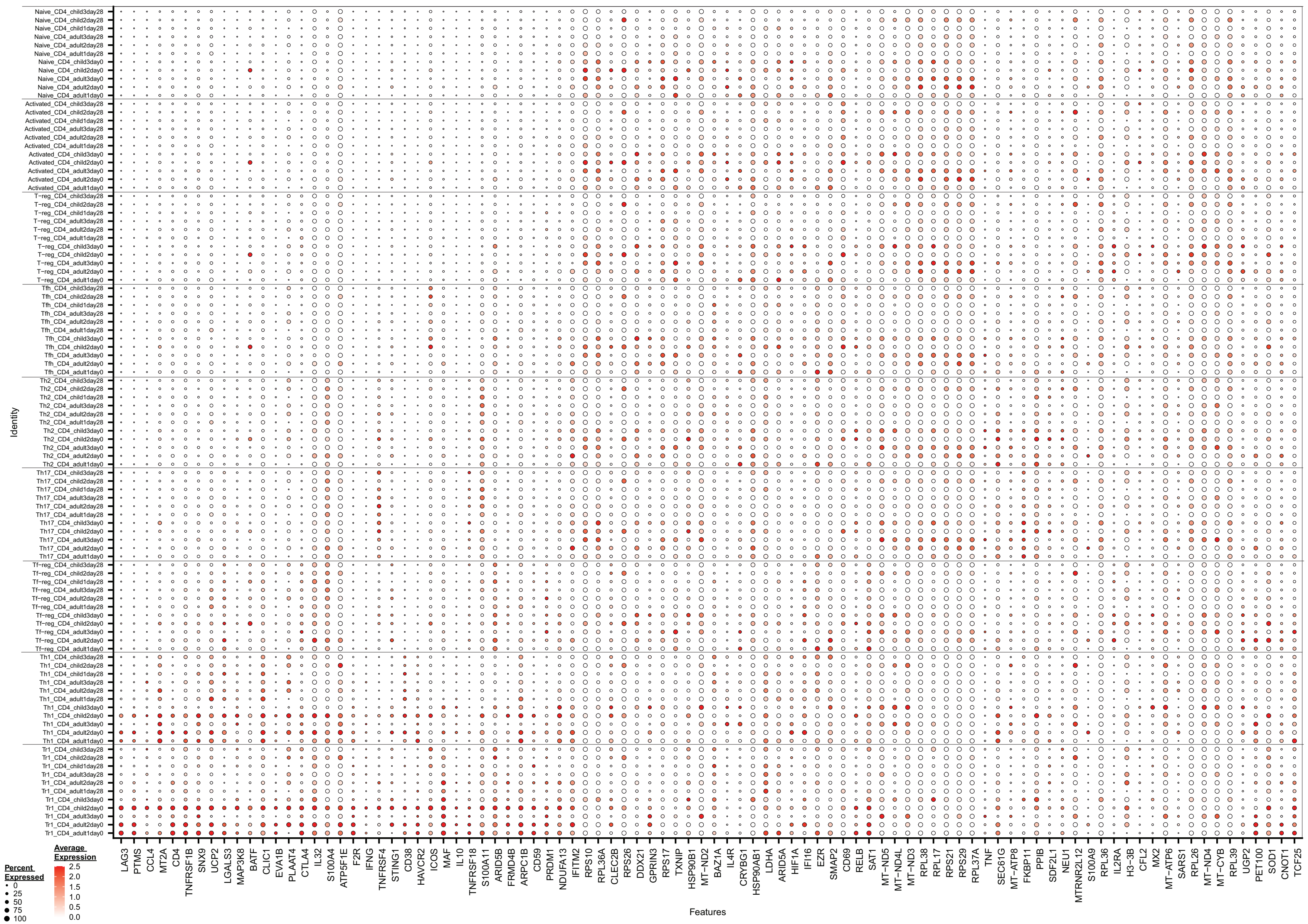

Supplementary Fig. 14: Malaria driven differential expressed genes in CD4 T cell subsets as average expression of each donor at day 0 and day 28.

Average expression level (white to red) and percentage of expression (dot size) in cell population of genes in the individual at day 0 and day 28 from each cell cluster.

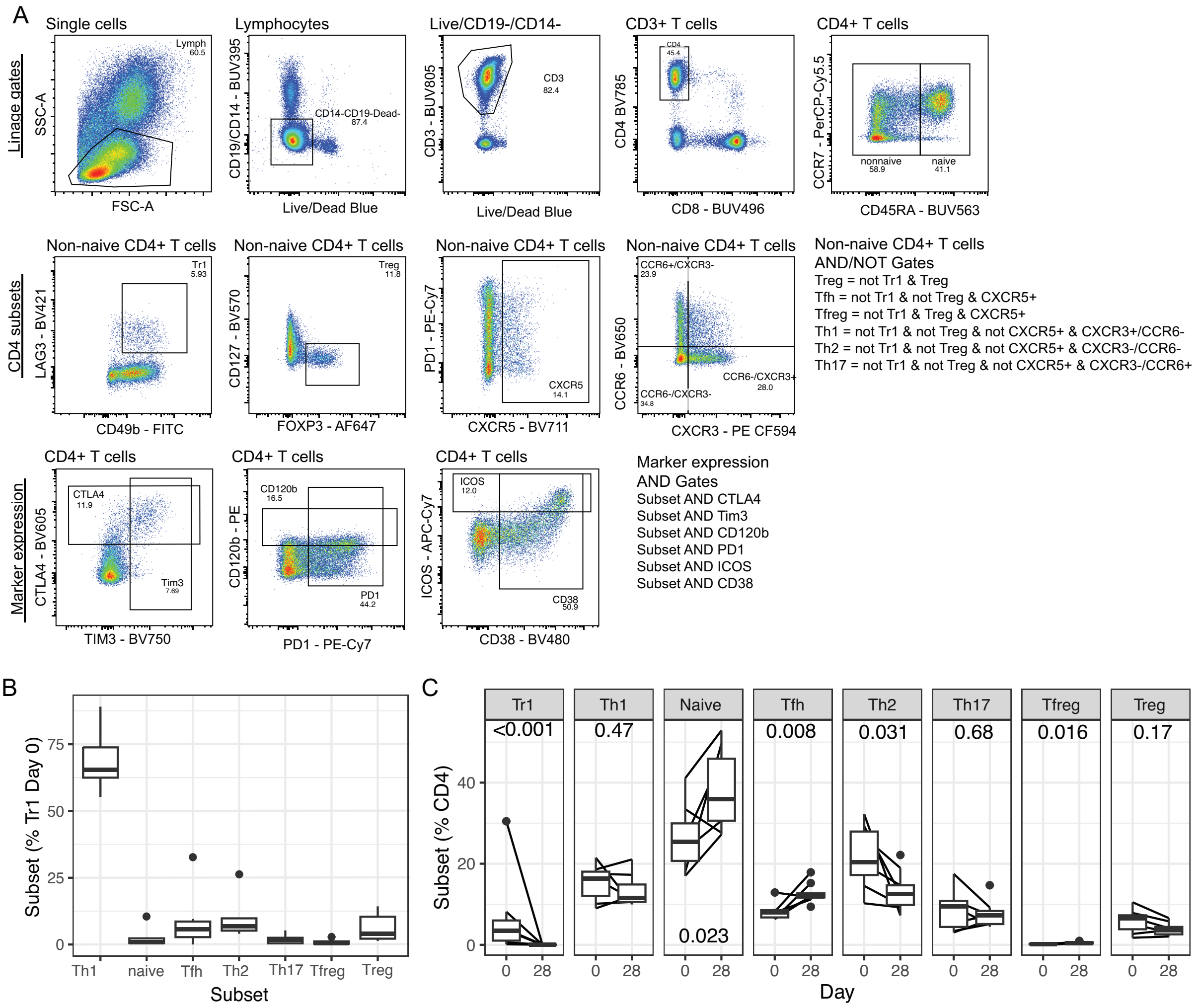

Supplementary Fig. 15: Flow cytometric analysis of CD4 T cells. PBMCs from individuals with acute *Plasmodium falciparum* infection (day 0, *n*=7) and 28-days post-infection (day 28 *n*=9), were analysed *ex vivo* to identify NK cell protein expression of DEGs. (**A**) Flow cytometry gating strategy to identify CD4 T cell subsets: Tr1, Th1, Naive, Tfh, Th2, Th17, Tfreg and Treg. (**B**) CD4 T cell subsets proportions of Tr1 cells. (**C**) CD4 T cell subsets proportion of total CD4 at day 0 and day 28. Boxplots show the median and IQR of volunteers, lines represent paired observations, group comparisons performed by Mann Whitney U test.

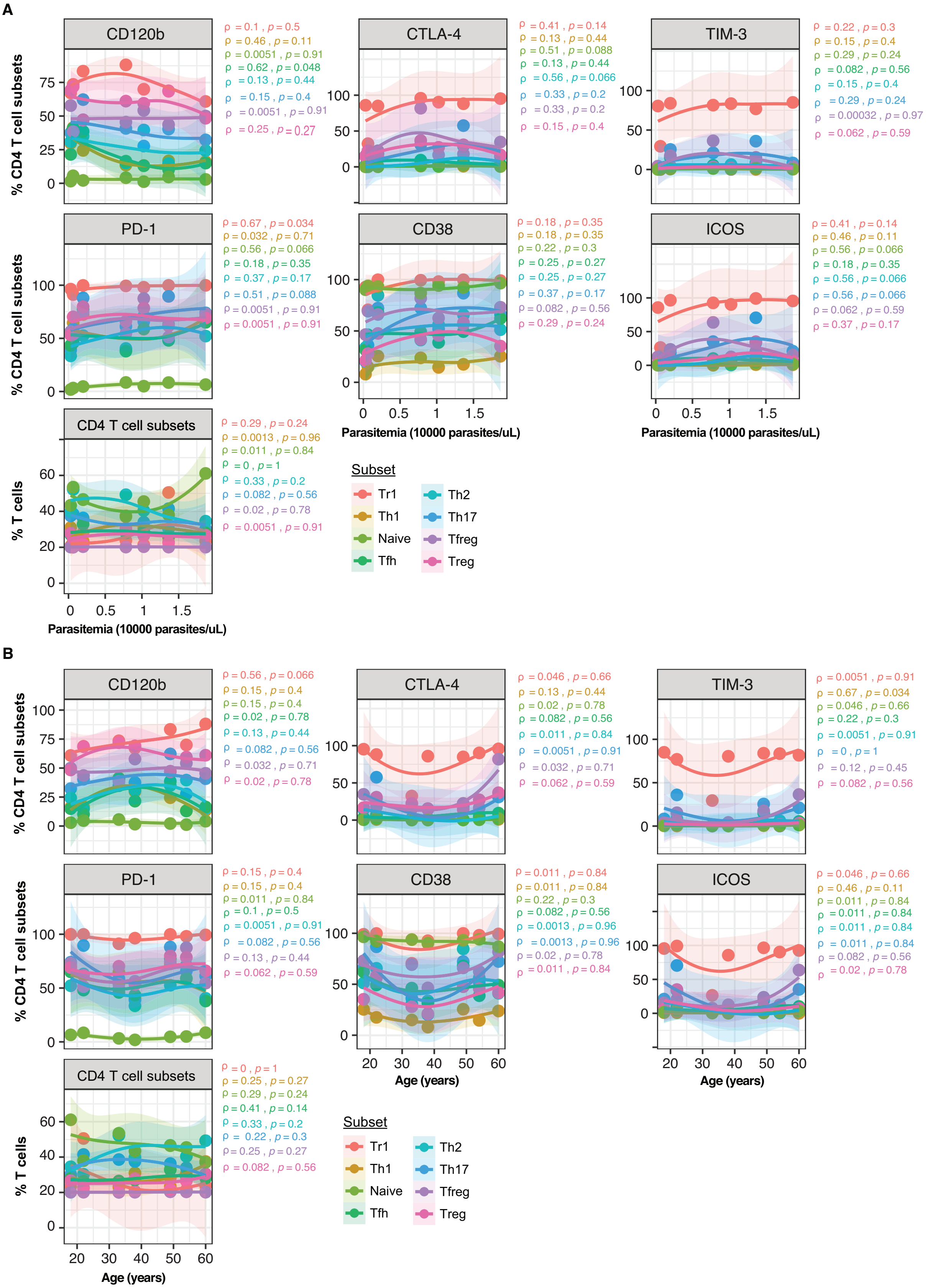

Supplementary Fig. 16: Association of blood parasitemia and age with CD4 T cell *ex vivo* phenotypes and frequencies. PBMCs from individuals with acute *Plasmodium falciparum* infection (day 0, n=11) were analysed *ex vivo*. CD4 T cell frequencies and marker expression associated with (**A**)

Plasmodium falciparum parasitemia (10000 parasites/μL) and (**B**) age (years). Scatterplot line performed by Loess regression, Spearman's rank correlation.

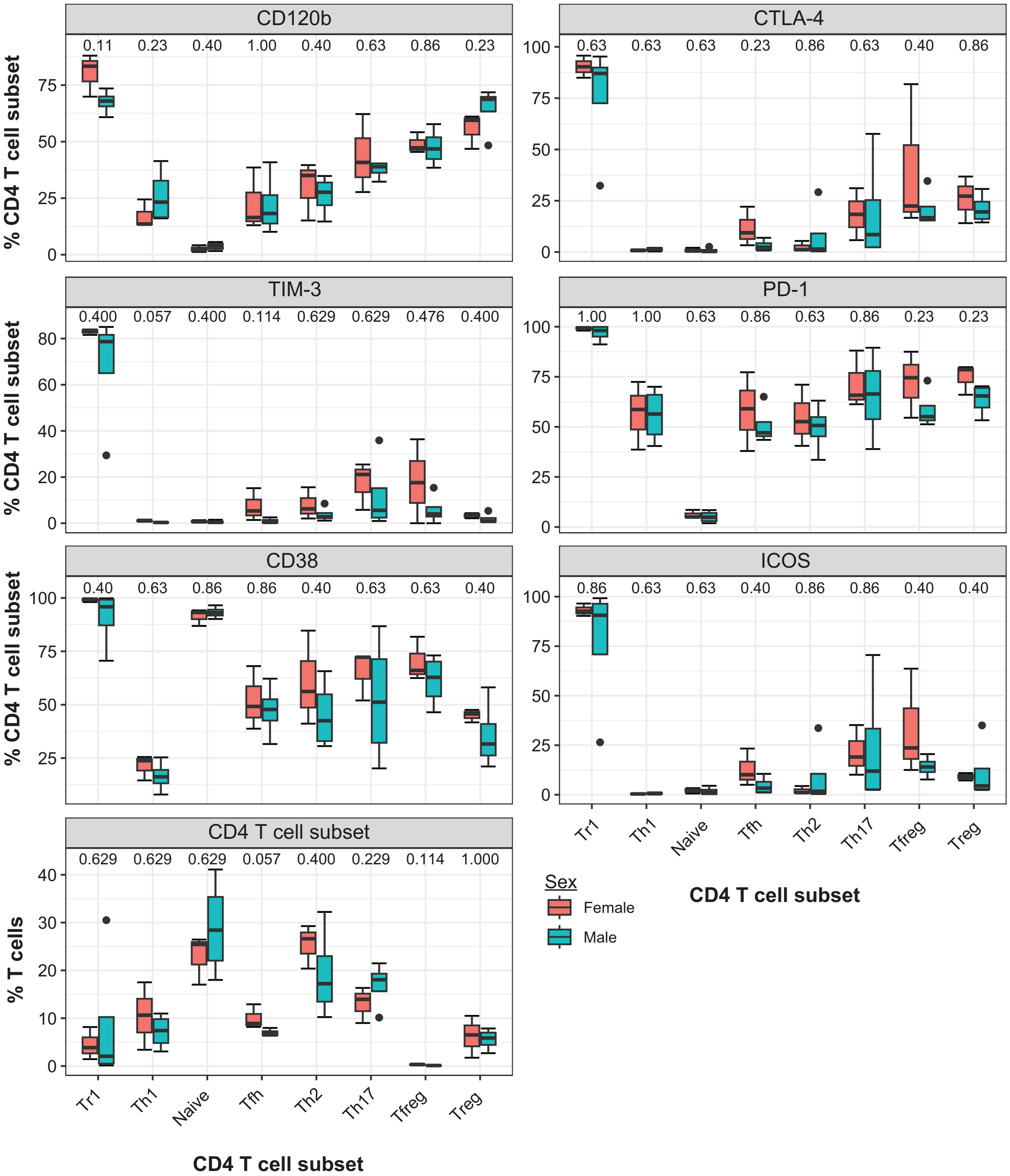

Supplementary Fig. 17: Association of sex with CD4 T cell *ex vivo* phenotypes and frequencies. PBMCs from individuals with acute *Plasmodium falciparum* infection (day 0, *n*=11) were analysed *ex vivo*. CD4 T cell frequencies and marker expression associated with sex Scatterplot line performed by Loess regression, Spearman's rank correlation. Boxplots show the median and IQR of volunteers, comparisons performed by Mann Whitney U test.

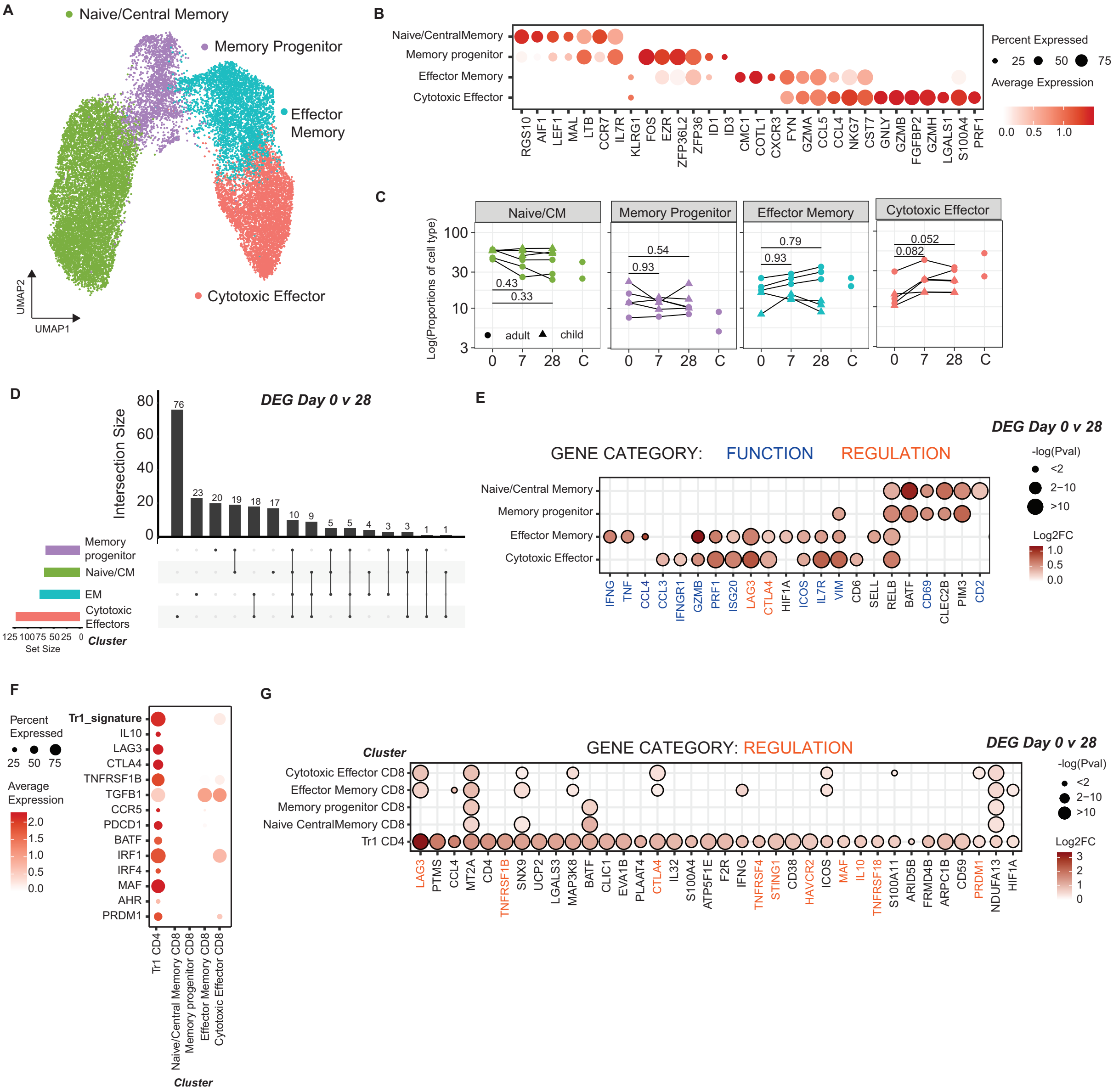

Supplementary Fig. 18: Activation and regulation of CD8 T cells during malaria. (**A/B**) Four subsets of CD8 T cells were identified based on unsupervised clustering and marker expression as Naive/Central Memory, Memory Progenitor, Effector Memory and Cytotoxic Effector. (**C**) Relative proportions of identified of CD8 T cell subsets within scRNAseq data during malaria infection (day 0), 7 days and 28 days post treatment, and in healthy uninfected individuals (C) (*n*=5 day 0, *n*=6 day 7, *n*=6 day 28, *n*=2 C). P-value indicated is calculated by Mann-Whitney U test between day 0 and indicated time points. (**D**) Numbers of upregulated DEGs in CD8 T cell subsets day 0 compared to day 28. The number of shared and subset specific DEGs indicated. (**E**) Top 10 upregulated DEGs in each CD8 T cells subsets with p ≤0.05 and known function. Genes with inflammatory/cytotoxic function and

roles in regulation are indicated. Log transformed p values generated with the Benjamini-Hochberg procedure to control false discovery rate (FDR). (**F**) Average expression of canonical Tr1 genes and Tr1_signature in Tr1 CD4 T cells and CD8 T cell subsets. (**G**) Top 20 DEGs upregulated during acute malaria in Tr1 CD4 directly compared with differential expression in non-Tr1 CD4 T cells and CD8 T cell clusters.

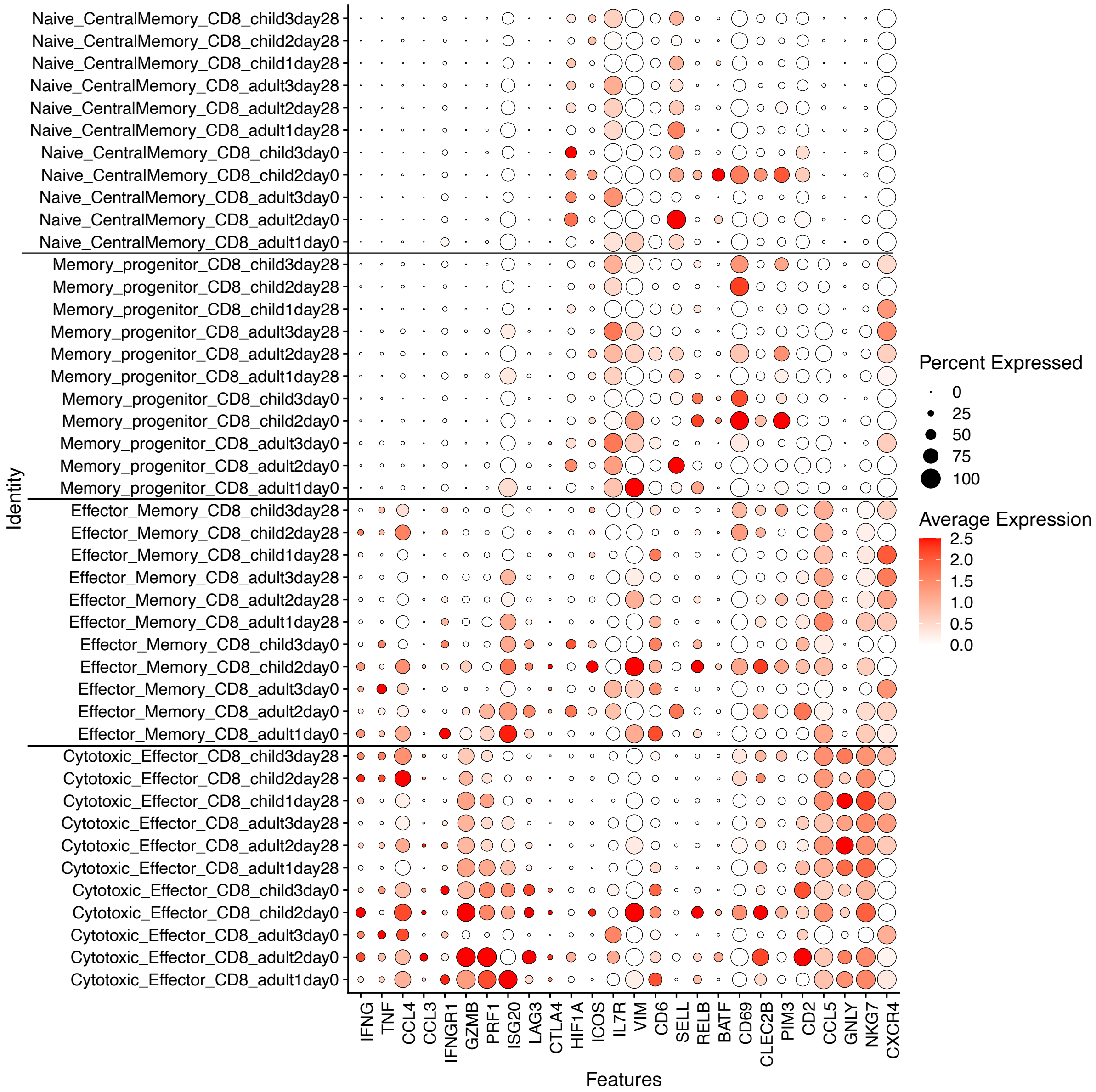

Supplementary Fig. 19: Malaria driven differential expressed genes in CD8 T cell subsets as average expression of each donor at day 0 and day 28. Average expression level (white to red) and percentage of expression (dot size) in cell population of genes in the individual at day 0 and day 28 from each cell cluster.

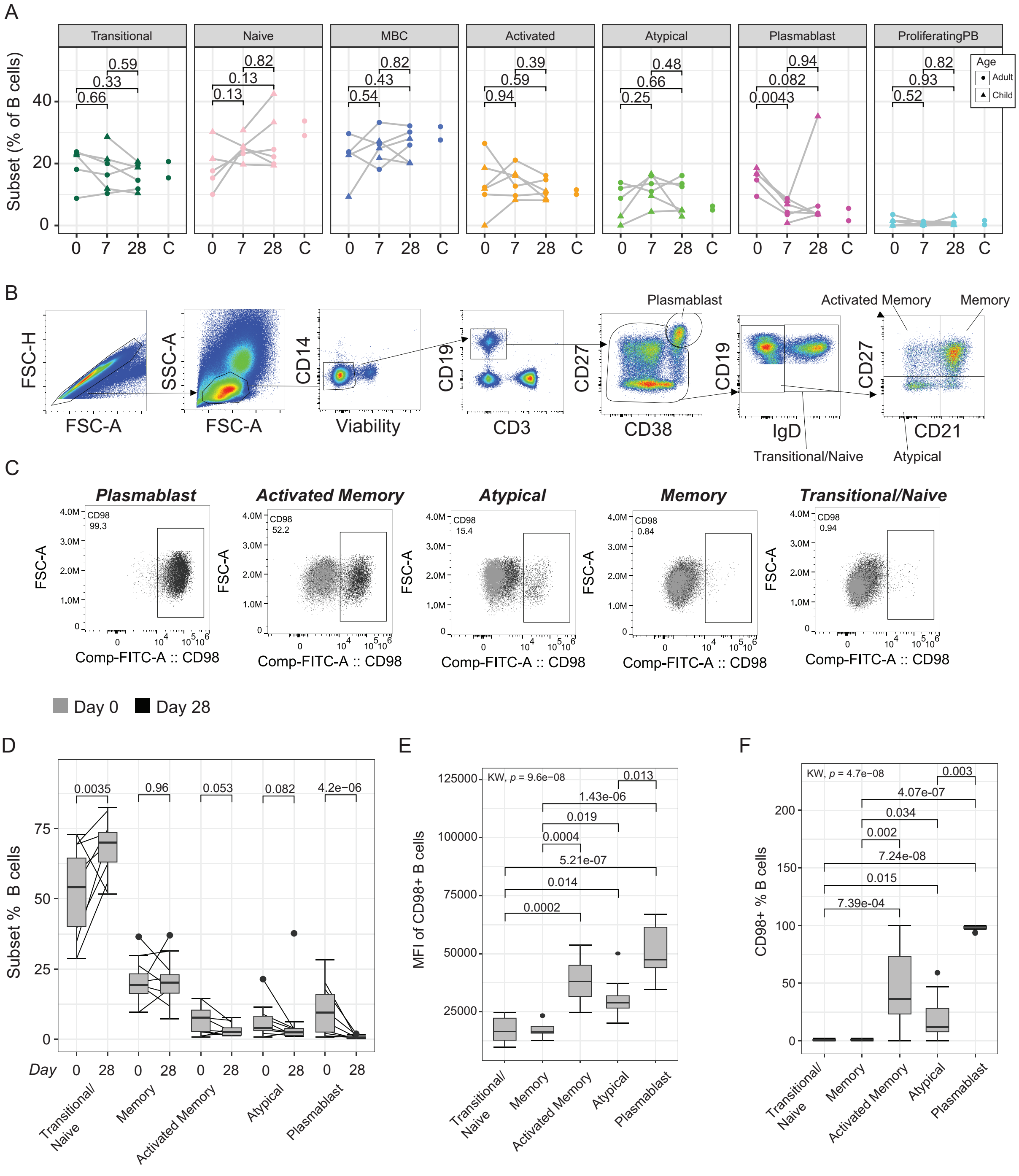

Supplementary Fig. 20: Proportional distribution of B cell subsets and CD98 protein expression. (**A**) Relative log10 proportions of identified of CD8 T cell subsets within scRNAseq data during malaria infection (day 0), 7 days and 28 days post treatment, and in healthy uninfected individuals (C) (*n*=5 day 0, *n*=6 day 7, *n*=6 day 28, *n*=2 C). (**B**) B cell subset gating strategy. (**C**) Representative FACS gating of CD98 expression on each B cell subset at day 0 versus day 28. (**D**) B cell subset proportion of total B cells at day 0 (n=11) and day 28 (n=17). P-values calculated by Mann Whitney U test. (**E**) MFI of

$CD98^+$ B cell subsets (*n*=5 day 0, *n*=8 day 28) and (**F**) CD98+ proportion of B cell subsets at day 0 (*n*=11 day 0). Kruskal-Wallis and post-hoc Dunn test (FDR adjusted) indicated.

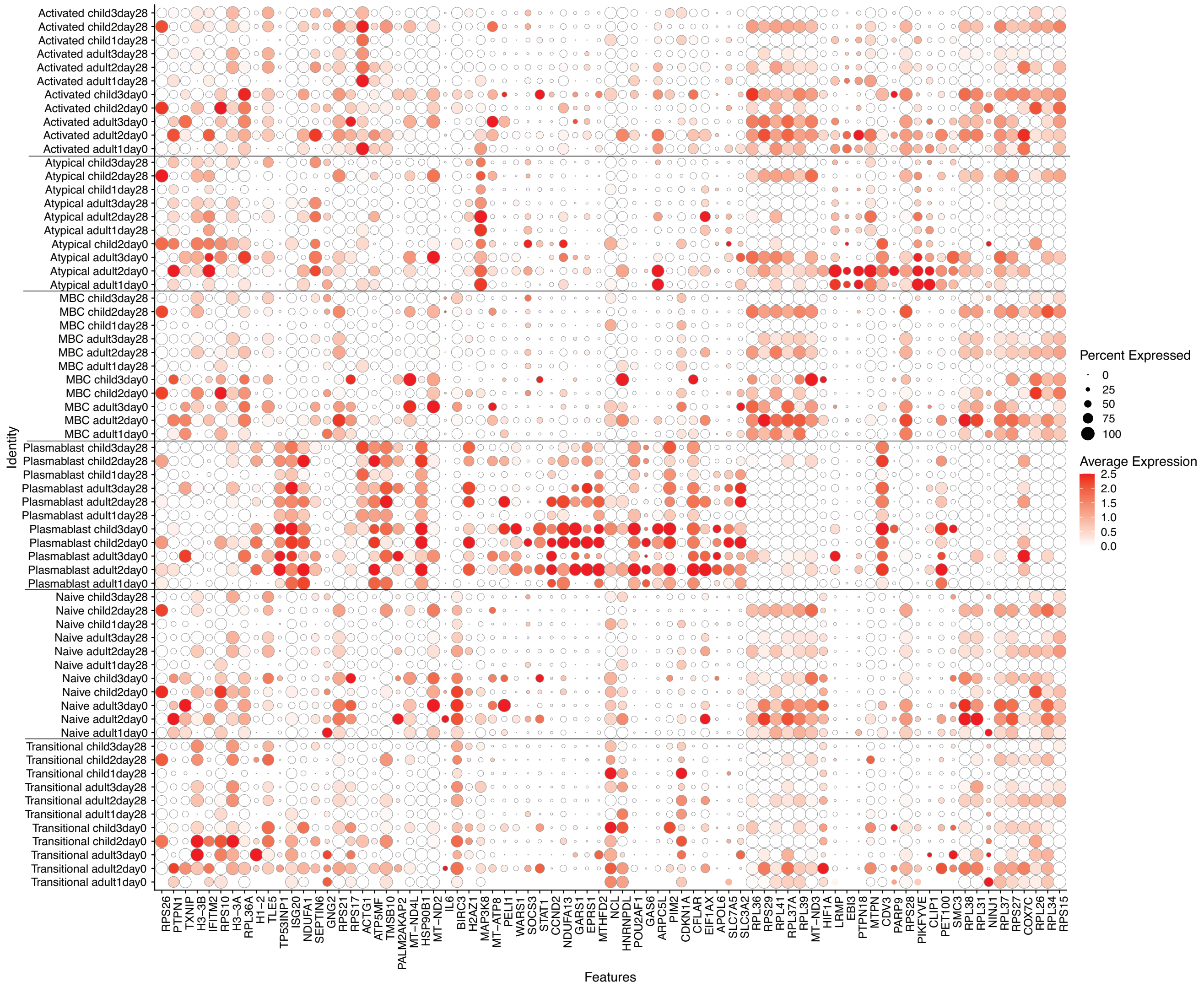

Supplementary Fig. 21: Malaria driven differential expressed genes in B cell subsets as average expression of each donor at day 0 and day 28. Average expression level (white to red) and percentage of expression (dot size) in cell population of genes in the individual at day 0 and day 28 from each cell cluster.

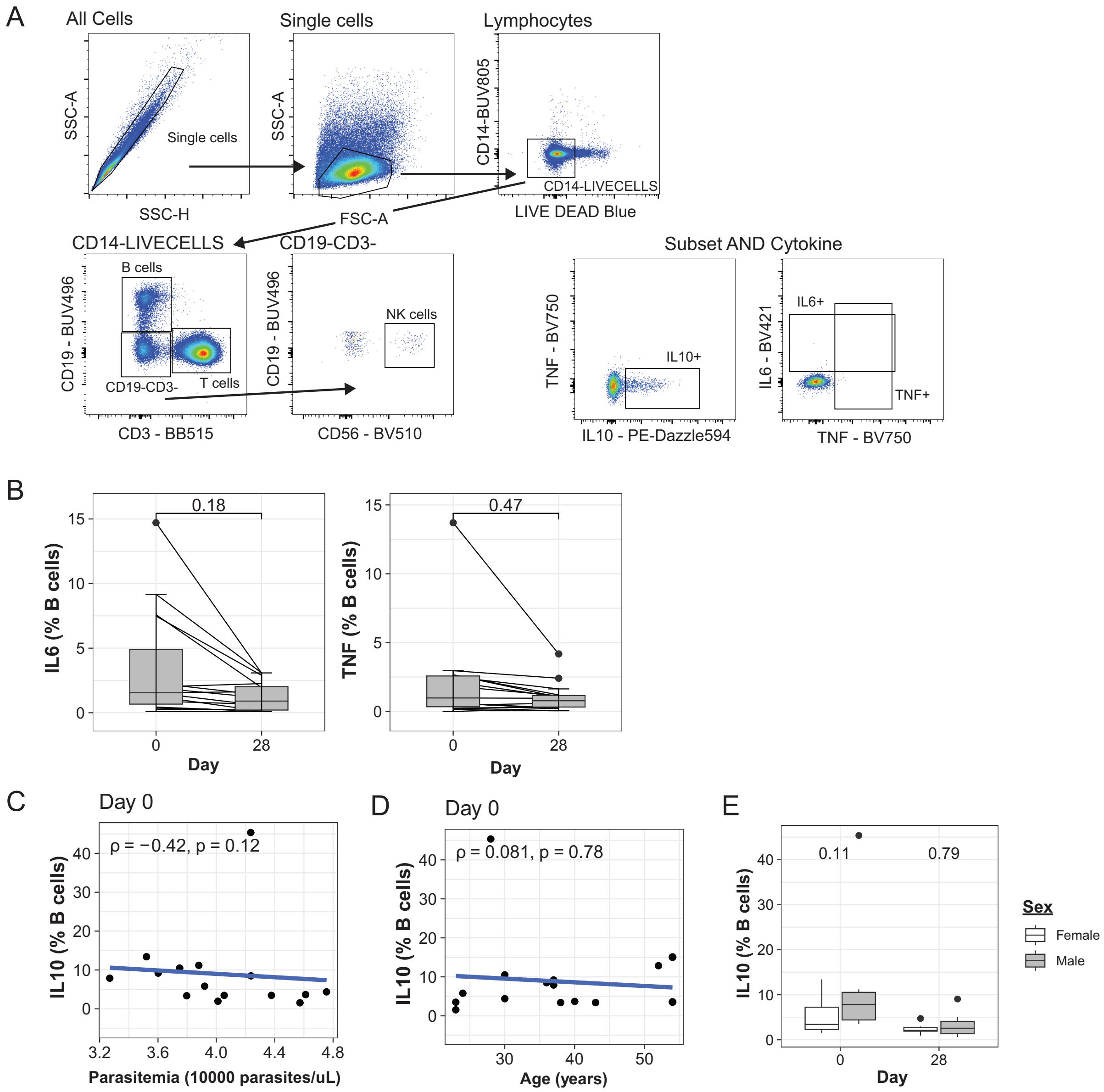

Supplementary Fig. 22: B cell *ex vivo* intracellular cytokine capture. To investigate key changes to B cell cytokine production, PBMCs from patients with malaria (day 0) and 28 days post treatment were rested *ex vivo* for 4 hours and cytokine captured by addition of Monensin then measured by flow cytometry. (**A**) B cell gating strategy and representative FACS gating of IL10, IL6 and TNF intracellular cytokine expression. (**B**) IL6 and TNF expression in B cells at day 0 (*n*=15) and day 28 (*n*=14). Frequency of IL10 positive B cells linear association with (**C**) *Plasmodium falciparum* parasitemia (10000 parasites/μL), (**D**) age (years) and (**E**) sex. Scatterplot line performed by linear regression, Spearman's rank correlation. Box plots show the median and IQR of volunteers, group comparisons performed by Mann Whitney U test.

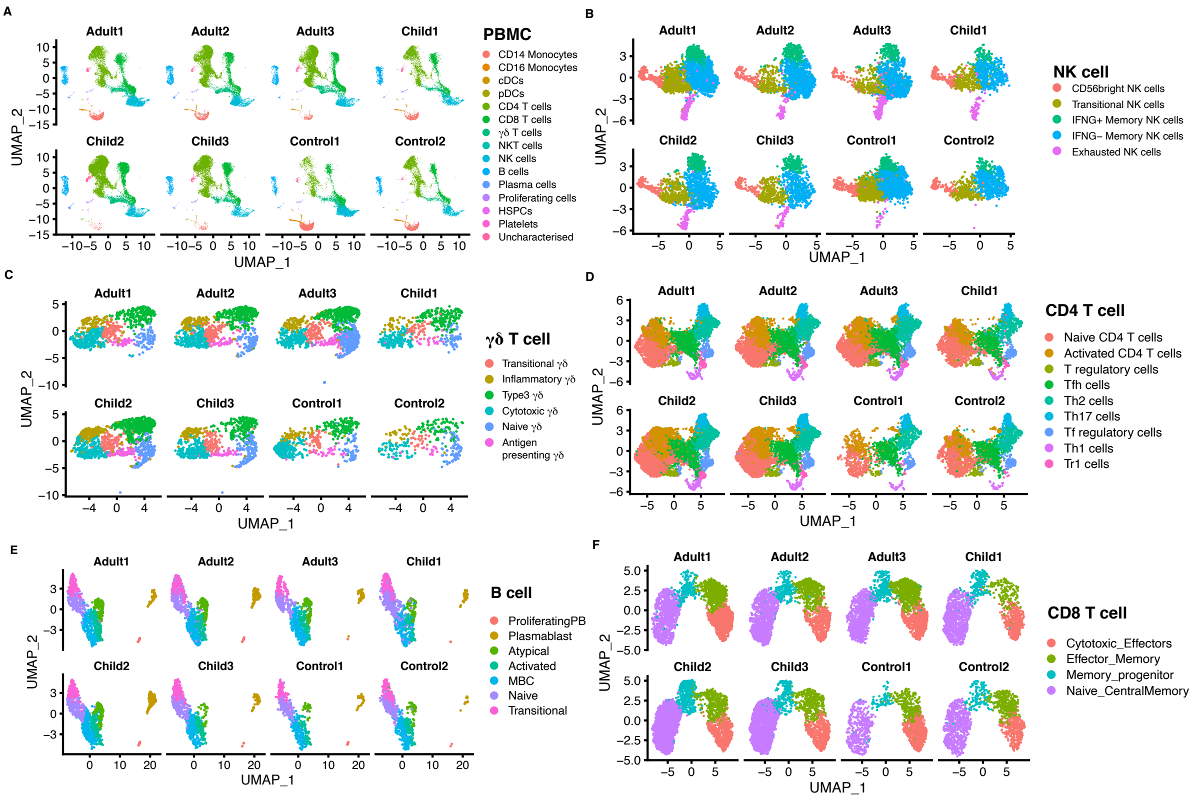

Supplementary Fig. 23: UMAP of PBMC and cell subset clusters by individual donor

UMAP of total PBMC analysis (**A**) and subclustered cells [NK cells, (**B**), γδ T cells (**C**), CD4 T cells (**D**), B cells (**E**), and CD8 T cells (**F**)], split by each individual donor.

Supplementary Table 1: Patient characteristics.

10XID	ID	Study Assay	Day	Age	Sex	Enrolment Date	Previous Malaria	Previous Malaria in last 2 months	Treatments Recieved	Parasite/uL (Enrol)	Fever	Body Temperature	Days with Fever	White blood cells (WBC) 10^3/uL	Haematocrit (HCT) %	Anaemia	Liver palpable	Spleen palpable	Mosquito Net Use	CMV Status
I/Q/J	adult2	scRNAseq	0/7/28	22	Female	17/06/15	No	NA	ASMQ	2522	Yes	38.5	4	8.7	44.4	No	No	No	NA	Positive
A/B/C	adult1	scRNAseq	0/7/28	21	Male	18/06/15	No	NA	ASMQ	30463	Yes	37.3	7	8.2	42.3	No	No	No	NA	Positive
O/T/P	child3	scRNAseq	0/7/28	13	Male	18/06/15	No	NA	ASMQ	1459	Yes	37.3	2	6.7	37.7	No	No	No	NA	Positive
M/S/N	child2	scRNAseq	0/7/28	8	Male	6/07/15	No	NA	ASMQ	40662	Yes	37.5	5	6.7	34	No	No	No	NA	Positive
		monoB-P	0	23	Female	2/05/15	No	NA	ART & AL	37404	Yes	39.7	2	7.2	39.4	No	No	No	NA	Positive
E/F/G	child1	scRNAseq	0/7/28	6	Male	18/06/15	No	NA	ART & AL & PQ	146388	Yes	40.3	3	3.3	25.1	No	No	No	NA	Positive
K/R/L	adult3	scRNAseq, gdbnk-P, CD4-P	0/7/28	24	Male	18/06/15	No	NA	AL & PQ	34	Yes	36.8	3	6.8	42.6	No	No	No	NA	Positive
D	control1	scRNAseq	NA	27	Male	6/07/11	NA	NA	NA	NA	No	36	NA	NA	NA	NA	NA	NA	always	Positive
H	control2	scRNAseq	NA	20	Male	13/07/11	NA	NA	NA	NA	No	36.8	NA	NA	NA	NA	NA	NA	never	Positive
	adult4	gdbnk-P	0	45	Male	26/04/11	No	NA	AL	81062	Yes	40	14	8.4	46.1	No	No	No	always	Positive
	adult5	gdbnk-P	0	37	Female	27/04/11	No	NA	ART & AL	32267	Yes	40	7	6	36.7	No	No	Hackett score 1	never	Positive
	adult6	gdbnk-P	0	39	Male	29/04/11	No	NA	AL	1508	Yes	37.5	2	5.2	40.3	No	No	No	never	Positive
	adult7	gdbnk-P	28	49	Male	9/05/11	No	NA	CQ & PQ & AL	NA	NA	NA	NA	NA	NA	NA	NA	NA	never	Positive
	adult8	gdbnk-P	28	54	Male	9/05/11	No	NA	CQ & ART & AL & PQ	NA	NA	NA	NA	NA	NA	NA	NA	NA	never	Positive
	adult9	gdbnk-P	0/28	54	Female	6/06/11	Yes	No	ART	27905	Yes	38.4	7	8.1	42.7	No	No	No	sometimes	Positive
	adult10	gdbnk-P ,monoBnk-P	0/28	36	Female	9/06/11	No	NA	ASMQ	17263	Yes	37.2	4	6.1	38.3	No	No	No	always	Positive
	adult11	monoBnk-P	0/28	54	Female	30/06/11	No	NA	ASMQ	10251	Yes	38.2	3	5.1	34.8	No	No	No	always	Positive
	adult12	gdbnk-P	28	39	Male	8/07/11	No	NA	AL & PQ	NA	NA	NA	NA	NA	NA	NA	NA	NA	sometimes	Positive
	adult13	gdbnk-P	28	46	Female	11/07/11	Yes	No	ART & AL	NA	NA	NA	NA	NA	NA	NA	NA	NA	always	Positive
	adult14	monoBnk-P	0/28	40	Male	11/07/11	Yes	No	AL	40895	Yes	38.7	8	8.7	40.7	No	No	No	never	Positive
	adult15	monoBnk-P	0	37	Male	3/09/11	Yes	No	AL	3990	Yes	36	4	7.6	38.3	No	No	No	never	Positive
	adult16	monoBnk-P	0/28	30	Male	21/09/11	No	NA	AL	56922	Yes	39.6	14	7.8	41.2	No	No	No	always	Positive
	adult17	monoBnk-P	28	39	Male	16/10/11	No	NA	AL	NA	NA	NA	NA	NA	NA	NA	NA	NA	always	Positive
	adult18	monoBnk-P	0/28	37	Male	19/11/11	No	NA	ART & AL	1862	Yes	38.4	4	5.1	38.7	NA	No	No	never	Positive
	adult19	monoBnk-P	0/28	43	Female	15/12/11	Yes	No	AL & PQ	11290	Yes	37	7	6.7	27.9	NA	Yes	No	never	Positive
	adult20	monoBnk-P	0/28	24	Male	24/12/11	No	NA	ART & AL	8321	Yes	39.1	8	5.3	35.4	NA	No	No	never	Positive
	adult21	monoBnk-P	0/28	38	Female	9/01/12	No	NA	AL	6254	Yes	39.9	2	5.3	38.8	NA	Yes	No	always	Positive
	adult22	monoBnk-P	0/28	28	Male	14/02/12	No	NA	ART & AL	17208	Yes	39	7	7.2	38.8	NA	Yes	No	never	Positive
	adult23	monoBnk-P	0/28	23	Male	6/03/12	No	NA	ART & AL	23868	Yes	37.6	10	5.1	29.1	No	Yes	No	always	Positive
	adult24	monoBnk-P	0/28	54	Female	22/03/12	No	NA	ART & AL	3317	Yes	37.5	3	9.9	29.5	NA	Yes	No	never	Positive
	adult25	gdbnk-P	28	16	Male	2/06/12	Yes	No	AL & CQ & PQ	NA	NA	NA	NA	NA	NA	NA	NA	NA	never	Positive
	adult26	gdbnk-P	28	21	Female	9/08/12	Yes	No	ART & AL & PQ	NA	NA	NA	NA	NA	NA	NA	NA	NA	never	Positive
	adult27	monoBnk-P	0/28	52	Male	7/09/12	Yes	No	ART & AL	7563	Yes	36.4	3	5.5	39.2	NA	No	No	never	Positive
	adult28	monoBnk-P	0/28	30	Male	3/12/12	No	NA	AL	5600	Yes	37.8	9	6.4	37.3	NA	No	No	never	Positive
	adult29	gdbnk-P, CD4-P	0/28	54	F	30/6/11	No	NA	ASMQ	10251	Yes	38.2	3	5.1	34.8	No	No	No	always	Positive
	adult30	gdbnk-P, CD4-P	0/28	33	M	18/11/11	No	NA	AL	633	Yes	37.4	1	5.5	39.8	NA	Yes	No	never	Positive
	adult31	gdbnk-P, CD4-P	0/28	38	M	30/12/11	No	NA	ART & AL	295	Yes	37.1	3	7.4	50.3	NA	Yes	No	never	Positive
	adult32	gdbnk-P, CD4-P	0/28	18	M	9/2/12	No	NA	ART & AL	18676	Yes	38.6	6	9.2	41	No	Yes	Yes	never	Positive
	adult33	gdbnk-P, CD4-P	0/28	54	F	30/6/11	No	NA	ASMQ	10251	Yes	38.2	3	5.1	34.8	No	No	No	always	Positive
	adult34	gdbnk-P, CD4-P	0/28	49	F	13/3/12	No	NA	AL	1988	Yes	38.1	6	5.3	27.5	NA	No	No	sometimes	Positive
	adult35	gdbnk-P, CD4-P	0/28	60	F	28/5/12	No	NA	ART & AL	7816	Yes	37.6	2	7.7	44.8	No	Yes	No	never	Positive
	adult36	gdbnk-P, CD4-P	0/28	22	M	6/6/11	No	NA	ASMQ	13633	Yes	37.2	5	4.1	15.9	No	No	No	never	Positive
	adult37	gdbnk-P, CD4-P	28	59	M	14/5/11	No	NA	AL	NA	NA	NA	NA	NA	NA	NA	NA	NA	always	Positive

Characteristics of child and adult patients from malaria-endemic Sabah enrolled and treated at hospital sites, and additional healthy, uninfected malaria-endemic controls. Samples were used in assays including 10X scRNA-seq of whole PBMC (scRNAseq, Fig. 1-6, S1-3, S5, S6, S11-14 and S19-21), *ex vivo* cytokine capture assay of myeloid, B and NK cells (monoBnk-P, Fig. 2 & 6, S4, S7, S22 and S23), *ex vivo* surface phenotyping of γδ T cells (gdbnk-P), B cells (gdbnk-P), NK cells (gdbnk-P) and CD4 T cells (CD4-P). Previous malaria is self-reported. Abbreviations: UM= Uncomplicated malaria, ASMQ = artesunate-mefloquine, ART = intravenous artesunate, AL = artemether-lumefantrine, PQ = primaquine, CQ= chloroquine, QEH = Queen Elizabeth Hospital, Kota Kinabalu.

Supplementary Table 2: Cell contributions to each PBMC subset by individual

	Day 0							Day 7							Day 28							Control	
	**Total**	child1	child2	child3	adult1	adult2	adult3	**Total**	child1	child2	child3	adult1	adult2	adult3	**Total**	child1	child2	child3	adult1	adult2d	adult3	adultc1	adultc2
CD14 Monocytes	**729**		54	48	2	619	6	**1431**	289	38	34	895	96	79	**923**	430	41	109	101	25	217	2599	894
CD16 Monocytes	**95**		6	4	1	84	0	**248**	68	2	8	121	16	33	**229**	104	0	10	46	20	49	477	353
cDCs	**27**		0	1	0	26	0	**72**	20	0	2	26	10	14	**62**	24	0	18	1	1	18	155	79
pDCs	**54**		15	8	0	27	4	**81**	3	7	12	31	4	24	**100**	46	27	4	3	11	9	71	62
CD4 T cells	**9132**		3685	1599	1226	1342	1280	**15330**	798	4162	2696	1320	2986	3368	**14734**	2437	3153	2470	1916	2246	2512	1868	3542
CD8 T cells	**2965**		1323	312	679	378	273	**6816**	483	1422	1445	956	1301	1209	**7422**	1403	1198	1280	1375	780	1386	1708	1640
γδ T cells	**871**		409	71	145	111	135	**2271**	155	458	327	350	552	429	**2881**	423	561	388	422	363	724	824	330
NKT cells	**57**		5	1	49	1	1	**269**	9	22	3	55	134	46	**466**	135	25	53	132	64	57	278	98
NK cells	**987**		315	124	395	95	58	**4719**	303	931	540	1161	1074	710	**4117**	898	297	364	1110	567	881	3028	1093
B cells	**623**		220	35	180	159	29	**2767**	373	504	311	347	743	489	**2515**	888	213	415	368	241	390	518	833
Plasmablasts	**124**		49	8	22	40	5	**191**	4	44	26	18	77	22	**241**	34	133	17	29	11	17	40	15
Proliferating cells	**163**		79	22	23	34	5	**151**	18	29	33	21	27	23	**119**	37	18	21	14	13	16	51	22
HSPCs	**64**		40	13	7	3	1	**134**	5	17	13	70	22	7	**50**	18	16	2	4	3	7	15	3
Platelets	**7**		2	4	0	1	0	**21**	5	1	0	4	4	7	**13**	1	3	2	3	2	2	14	8
Uncharacterised	**294**		72	114	17	18	73	**318**	20	89	60	60	36	53	**414**	83	148	56	50	42	35	106	55

Supplementary Table 3: Cell contributions to each NK cell subset by individual

	Day 0							Day 7							Day 28							Control	
	**Total**	child1	child2	child3	adult1	adult2	adult3	**Total**	child1	child2	child3	adult1	adult2	adult3	**Total**	child1	child2	child3	adult1	adult2d	adult3	adultc1	adultc2
CD56++	**85**		33	6	38	8	0	**395**	35	93	64	86	81	36	**330**	117	25	33	68	40	47	231	106
IFNy- Adaptive	**439**		147	67	139	50	36	**2144**	111	361	201	491	609	371	**1984**	320	151	157	568	356	432	1625	611
IFNy+ Adaptive	**161**		43	31	63	12	12	**607**	26	135	65	150	147	84	**527**	62	59	66	122	89	129	272	76
PD1+	**37**		3	0	31	0	3	**423**	44	112	43	78	54	92	**261**	39	11	11	66	11	123	31	4
Transitional	**248**		82	16	122	22	6	**1084**	84	221	157	350	155	117	**780**	173	44	84	271	64	144	808	281

Supplementary Table 4: Cell contributions to each γδ cell subset by individual

	Day 0							Day 7							Day 28							Control	
	**Total**	child1	child2	child3	adult1	adult2	adult3	**Total**	child1	child2	child3	adult1	adult2	adult3	**Total**	child1	child2	child3	adult1	adult2d	adult3	adultc1	adult2c
APC	**55**		38	5	2	4	6	**103**	4	37	20	7	24	11	**185**	18	24	22	37	22	62	36	16
Cytotoxic	**137**		71	9	33	11	13	**537**	48	93	82	108	150	56	**684**	111	96	111	129	114	123	411	68
Inflammatory	**100**		55	2	26	12	5	**248**	10	67	39	36	65	31	**337**	34	91	64	41	57	50	65	30
Naïve	**180**		81	23	16	19	41	**343**	12	83	61	21	43	123	**555**	52	67	69	46	47	274	64	86
Transitional	**83**		43	3	20	9	8	**334**	18	45	51	72	96	52	**261**	38	56	34	36	43	54	55	31
Type3	**292**		119	27	32	54	60	**658**	55	127	71	82	169	154	**762**	127	221	87	92	78	157	133	83

Supplementary Table 5: Cell contributions to each CD4 cell subset by individual

	Day 0							Day 7							Day 28							Control	
	**Total**	child1	child2	child3	adult1	adult2	adult3	**Total**	child1	child2	child3	adult1	adult2	adult3	**Total**	child1	child2	child3	adult1	adult2d	adult3	adultc1	adult2c
Activated	**2381**		1092	527	283	198	281	**3212**	173	1060	667	229	510	573	**3180**	513	948	543	356	430	390	273	755
Naïve	**2924**		1296	568	296	347	417	**5354**	265	1751	932	353	913	1140	**4839**	808	1077	861	536	744	813	355	948
T-reg	**172**		88	32	12	27	13	**275**	19	46	97	26	51	36	**310**	68	58	55	35	59	35	44	96
Tf-reg	**329**		112	41	40	104	32	**499**	31	80	86	73	106	123	**472**	76	77	82	60	76	101	118	127
Tfh	**1473**		453	181	265	339	235	**2677**	126	673	387	250	581	660	**2744**	432	514	380	441	484	493	439	721
Th1	**134**		59	6	24	32	13	**295**	22	97	40	30	47	59	**278**	63	34	48	41	38	54	32	31
Th17	**512**		122	94	84	92	120	**917**	70	95	162	82	246	262	**1016**	236	121	181	134	126	218	273	425
Th2	**998**		368	140	174	161	155	**1778**	78	315	288	210	419	468	**1704**	206	309	298	252	264	375	319	427
Tr1	**209**		95	10	48	42	14	**323**	14	45	37	67	113	47	**191**	35	15	22	61	25	33	15	12

Supplementary Table 6: Cell contributions to each CD8 cell subset by individual

	**Total**	child1	child2	child3	adult1	adult2	adult3	**Total**	child1	child2	child3	adult1	adult2	adult3	**Total**	child1	child2	child3	adult1	adult2	adult3	**Total**	adultc1	adultc2
Cytotoxic Effector	**429**		136	44	174	41	34	**1495**	107	219	228	382	282	277	**1700**	297	184	199	426	167	427	**1211**	824	387
Effector Memory	**405**		109	51	112	65	68	**1302**	62	215	184	226	271	344	**1461**	115	130	159	384	185	488	**697**	313	384
Memory progenitor	**464**		293	38	45	45	43	**758**	64	168	138	68	167	153	**881**	173	255	127	108	80	138	**218**	80	138
Naive/Central Memory	**1567**		775	179	262	224	127	**3169**	230	816	893	237	562	431	**3176**	729	627	790	362	336	332	**1024**	401	623

Supplementary Table 7: Cell contributions to each B cell subset by individual

	Day 0							Day 7							Day 28							Control	
	**Total**	child1	child2	child3	adult1	adult2	adult3	**Total**	child1	child2	child3	adult1	adult2	adult3	**Total**	child1	child2	child3	adult1	adult2d	adult3	adultc1	adult2c
Transitional	**158**		61	10	48	36	3	**505**	108	65	72	73	134	53	**462**	190	36	81	58	49	48	86	175
Naive	**128**		58	13	31	20	6	**724**	116	108	86	91	204	119	**838**	392	67	144	79	56	100	162	286
MBC	**180**		61	4	48	59	8	**766**	82	149	84	66	215	170	**686**	185	70	121	103	76	131	178	234
Activated	**94**		32	8	25	20	9	**397**	31	91	54	77	79	65	**303**	75	30	48	64	26	60	64	85
Atypical	**63**		8	0	28	24	3	**375**	36	91	15	40	111	82	**226**	46	10	21	64	34	51	28	53
Plasmablast	**110**		45	8	19	33	5	**175**	3	43	23	13	71	22	**221**	33	122	16	25	9	16	31	13
ProliferatingPB	**14**		4	0	3	7	0	**16**	1	1	3	5	6	0	**20**	1	11	1	4	2	1	9	2

Supplementary Table 8: Antibodies for *ex vivo* cell phenotyping comparison of scRNAseq samples

Target	Fluorochrome	Clone	Manufacturer	Catalogue number	Dilution
Surface Staining					
CD16	BUV395	3G8	BD	563785	1/500
CD45RA	BUV563	HI100	BD	565702	1/1000
CD4	BUV737	SK3	BD	612748	1/250
CD27	BV421	M-T271	BD	562513	1/250
CD123	PacBlue	6H6	Biolegend	306043	1/50
CD56	BV510	HCD56	Biolegend	318340	1/50
CD127	BV570	A019D5	Biolegend	351307	1/80
CD11c	BV650	Bu15	Biolegend	337238	1/500
CD8	BV711	RPA-T8	BD	563677	1/1000
CD19	BV750	HIB19	Biolegend	302261	1/500
HLA-DR	BV785	L243	Biolegend	307642	1/250
TCR γδ	FITC	B1	Biolegend	331208	1/50
CD3	AF532	UCHT1	Invitrogen	58-0038-42	1/50
CD14	PerCP-Cy5.5	M5E2	Biolegend	301824	1/100
ICOS	PE	DX29	BD	557802	1/100
CD86	PE-Daz594	IT2.2	Biolegend	305434	1/250
CXCR5	PE-Cy7	J252D4	Biolegend	356924	1/250
CD38	AF647	HIT2	Biolegend	303514	1/500
CD25	APC-R700	2A3	BD	565106	1/250
Vδ2	APC/Fire 750	B6	Biolegend	331420	1/500

Supplementary Table 9: Antibodies for *ex vivo* cytokine analysis

Antibody	Fluorochrome	Clone	Manufacturer	Catalogue number	Dilution
Surface staining					
CD19	BUV496	SJ25C1	BD	612938	1/100
CD64	BUV737	10.1	BD	564425	1/50
CD14	BUV805	M5E2	BD	612902	1/50
CD86	BV480	2331	BD	566131	1/50
CD3	BB515	UCHT1	BD	564466	1/100
CD56	BV510	HCD56	Biolegend	318340	1/50
CD33	BV570	WM53	Biolegend	303417	1/50
CD123	BV605	6H6	Biolegend	306026	1/100
CD11c	BV650	B915	Biolegend	117310	1/200
HLA-DR	BV785	L243	Biolegend	307642	1/100
CCR2	PerCPCy5.5	K036C2	Biolegend	357204	1/50
Vδ2	APC	B6	Biolegend	331418	1/50
CD16	AF700	3C78	Biolegend	302026	1/100
CD1c	APC-FIRE	L161	Biolegend	331545	1/30
Intracellular staining					
Live/Dead	-	-	Invitrogen	L23105	1/5000
IFN-γ	BUV395	B27	BD	563563	1/25
IL-6	BV421	C8-6	BD	501119	1/100
IL-4	BV711	MD4–25D2	BD	564112	1/25
TNF	BV750	Mab11	BD	566359	1/100
IL-12	AF647	503-F7	BD	565023	1/25
IL-10	PE-Dazzle	JES–19F1	BD	506812	1/25
MCP-1	PE-Cy7	5D3-F7	Biolegend	502614	1/100
IFN-α	PE	LT27-295	Miltenyi	130-092-602	1/100
IL-1β	FITC	CRM56	Invitrogen	11-7018-42	1/50

Supplementary Table 10: Antibodies for γδ T, NK and B cell *ex vivo* phenotyping analysis

Antibody	Fluorochrome	Clone	Manufacturer	Catalogue number	Dilution
Surface staining					
CD366 (Tim-3)	BV605	F38-2E2	Biolegend	345017	1 in 25
CD223 (LAG-3)	BV711	11C3C65	Biolegend	369319	1 in 50
CD27	AF700	M-T271	BD	560611	1 in 100
Vδ1	APC	TS8.2	Invitrogen	17-5679-42	1 in 200
Vδ2	APC-Fire	B6	Biolegend	331420	1 in 100
CD19	BUV395	SJ25C1	BD	563549	1 in 100
CD56	BUV563	NCAM16.2	BD	612928	1 in 500
CD279 (PD1)	BUV615	EH12.1	BD	612991	1 in 50
CD16	BUV737	3G8	BD	612787	1 in 500
CD3	BUV805	SK7	BD	612893	1 in 200
CD57	BV421	NK-1	BD	563896	1 in 500
CD38	BV480	HIT2	BD	566137	1 in 500
CD86	BV510	2331 (FUN-1)	BD	563461	1 in 100
CD14	BV570	M5E2	Biolegend	301831	1 in 50
CD278 (ICOS)	BV650	DX29	BD	563832	1 in 50
HLA-DR	BV750	L243	Biolegend	307672	1 in 200
IgD	BV785	IA6-2	Biolegend	348242	1 in 200
CD98	FITC	MEM-108	Biolegend	315603	1 in 50
Intracellular staining					
LIVE/DEAD Fixable Blue Dead Cell Stain	-	-	Invitrogen	L23105	1 in 2500
Perforin	PE-CF594	δG9	BD	563763	1 in 5000
Granzyme-B	PerCP-Cy5.5	QA16A02	Biolegend	372212	1 in 500

Supplementary Table 11: Antibodies for CD4 *ex vivo* phenotyping analysis

Antigen	Fluorophore	Clone	Manufacturer	Cat #	Concentrations
Surface staining					
LAG3	BV421	11C3C65	Biolegend	369314	1 in 50
CD49b	FITC	eBIoY41B(Y418)	Invitrogen	11-0498-42	1in 100
CCR7	PerCP-Cy5.5	150503	BD	561144	1 in 25
CD14	BUV395	MΦP9	BD	563561	1 in 100
CD19	BUV395	SJ25C1	BD	563549	1 in 100
CD8	BUV496	RPA-T8	BD	612942	1 in 400
CD3	BUV805	SK7	BD	612893	1 in 50
CD38	BV480	HIT2	BD	566137	1 in 50
CD127	BV570	A019D5	Biolegend	351308	1 in 50
CCR6	BV650	11A9	BD	563922	1 in 50
CXCR5	BV711	J252D4	Biolegend	356934	1 in 50
TIM3	BV750	F38-2E2	Biolegend	345056	1 in 50
CD4	BV785	OKT4	Biolegend	317442	1 in 50
CD120b	PE	3G7A02	Biolegend	358404	1 in 50
CXCR3	PE-DAZ594	1C6/CXCR3	BD	562451	1 in 50
PD1	PE-CY7	EH12.1	BD	561272	1 in 50
ICOS	APC-CY7	C398.4A	Biolegend	313530	1 in 25
Intracellular staining					
CTLA4	BV605	BNI3	Biolegend	369610	1 in 50
FOXP3	AF647	206D	Biolegend	320114	1 in 50
LIVE/DEAD Fixable Blue Dead Cell Stain			Invitrogen	L23105	1 in 2500
