# Supplementary material for: Single cell transcriptomics shows that malaria promotes unique regulatory responses across multiple immune cell subsets
Source: Nat Commun. 2023 Nov 15;14:7387. doi: 10.1038/s41467-023-43181-7 (PMC10651914; doi:10.1038/s41467-023-43181-7)
Supplement: Supplementary file 3 — Description of Additional Supplementary Files [file 41467_2023_43181_MOESM3_ESM.pdf]

Description of Additional Supplementary Files

Title: Supplementary Data 1

Description: **PBMC cluster marker genes**

Title: Supplementary Data 2

Description: **PBMC cluster DEGs**

Title: Supplementary Data 3

Description: **NK subset marker genes**

Title: Supplementary Data 4

Description: **NK subset DEGs**

Title: Supplementary Data 5

Description: **gd T cell subset marker genes**

Title: Supplementary Data 6

Description: **gd T cell subset DEGs**

Title: Supplementary Data 7

Description: **CD4 T cell subset marker genes**

Title: Supplementary Data 8

Description: **CD4 T cell subset DEGs**

Title: Supplementary Data 9

Description: **CD8 T cell subset marker genes**

Title: Supplementary Data 10

Description: **CD8 T cell subset DEGs**

Title: Supplementary Data 11

Description: **B cell subset marker genes**

Title: Supplementary Data 12

Description: **B cell subset DEGs**
