# Supplementary material for: Single cell transcriptomics shows that malaria promotes unique regulatory responses across multiple immune cell subsets
Source: Nat Commun. 2023 Nov 15;14:7387. doi: 10.1038/s41467-023-43181-7 (PMC10651914; doi:10.1038/s41467-023-43181-7)
Supplement: Supplementary file 16 — Reporting Summary [file 41467_2023_43181_MOESM16_ESM.pdf]

nature portfolio

Corresponding author(s): Michelle Boyle

Last updated by author(s): Sep 27, 2023

Reporting Summary

Nature Portfolio wishes to improve the reproducibility of the work that we publish. This form provides structure for consistency and transparency in reporting. For further information on Nature Portfolio policies, see our Editorial Policies and the Editorial Policy Checklist.

Statistics

For all statistical analyses, confirm that the following items are present in the figure legend, table legend, main text, or Methods section.

n/a	Confirmed	
☐	☒	The exact sample size (*n*) for each experimental group/condition, given as a discrete number and unit of measurement
☐	☒	A statement on whether measurements were taken from distinct samples or whether the same sample was measured repeatedly
☐	☒	The statistical test(s) used AND whether they are one- or two-sided<br>*Only common tests should be described solely by name; describe more complex techniques in the Methods section.*
☐	☒	A description of all covariates tested
☐	☒	A description of any assumptions or corrections, such as tests of normality and adjustment for multiple comparisons
☐	☒	A full description of the statistical parameters including central tendency (e.g. means) or other basic estimates (e.g. regression coefficient) AND variation (e.g. standard deviation) or associated estimates of uncertainty (e.g. confidence intervals)
☐	☒	For null hypothesis testing, the test statistic (e.g. *F*, *t*, *r*) with confidence intervals, effect sizes, degrees of freedom and *P* value noted<br>*Give P values as exact values whenever suitable.*
☒	☐	For Bayesian analysis, information on the choice of priors and Markov chain Monte Carlo settings
☒	☐	For hierarchical and complex designs, identification of the appropriate level for tests and full reporting of outcomes
☐	☒	Estimates of effect sizes (e.g. Cohen's *d*, Pearson's *r*), indicating how they were calculated

Our web collection on statistics for biologists contains articles on many of the points above.

Software and code

Policy information about availability of computer code

Data collection	Single cell sequencing data was demultiplexed, aligned and quantified using Cell Ranger version 3.1.0 software (10x Genomics) against the human reference genome (GRCh38-3.0.0), with default parameters.<br><br>The code generated during this study for subsequent analysis of scRNAseq data is available at https://doi.org/10.5281/zenodo.8378626, https://github.com/MichelleBoyle/scRNAseq_malaria_2023
Data analysis	R_4.2.2, Rstudio_2022.07.2 Build 576, RColorBrewer_1.1-3, DESeq2_1.38.1, png_0.1-8, apeglm_1.20.0, pheatmap_1.0.12, reshape2_1.4.4, magrittr_2.0.3, edgeR_3.40.0, scater_1.26.1, scuttle_1.8.2, devtools_2.4.5, usethis_2.1.6, patchwork_1.1.2, RCurl_1.98-1.9, cowplot_1.1.1, scales_1.2.1, Matrix_1.5-1, forcats_0.5.2, stringr_1.5.0, dplyr_1.0.10, purrr_0.3.5, readr_2.1.3, tidyr_1.2.1, tibble_3.1.8, tidyverse_1.3.2, SeuratObject_4.1.3, Seurat_4.3.0, SingleCellExperiment_1.20.0, SummarizedExperiment_1.28.0 Biobase_2.58.0, GenomicRanges_1.50.1, GenomeInfoDb_1.34.4, IRanges_2.32.0, S4Vectors_0.36.1, BiocGenerics_0.44.0, MatrixGenerics_1.10.0, matrixStats_0.63.0, limma_3.54.0, UpSetR_1.4.0, ggpubr_0.5.0, readxl_1.4.1, ggplot2_3.4.0, viridis_0.6.2, viridisLite_0.4.1, plyr_1.8.8, ape_5.6-2, GSEA publicly available software (https://www.gsea-msigdb.org/gsea/index.jsp

For manuscripts utilizing custom algorithms or software that are central to the research but not yet described in published literature, software must be made available to editors and reviewers. We strongly encourage code deposition in a community repository (e.g. GitHub). See the Nature Portfolio guidelines for submitting code & software for further information.

Data

Policy information about availability of data

All manuscripts must include a data availability statement. This statement should provide the following information, where applicable:

- Accession codes, unique identifiers, or web links for publicly available datasets
- A description of any restrictions on data availability
- For clinical datasets or third party data, please ensure that the statement adheres to our policy

Single cell sequencing data was demultiplexed, aligned and quantified using Cell Ranger version 3.1.0 software (10x Genomics) against the human reference genome (GRCh38-3.0.0), with default parameters. Raw sequencing data and processed Cell Ranger outputs are found at GSE217930, https://www.ncbi.nlm.nih.gov/geo/query/acc.cgi?acc=GSE217930.

All data generated or analysed during this study are included in this published article and supplementary information files.
Source data are provided as a Source Data file.

The raw sequencing data and processed Cell Ranger outputs used in this study have been deposited in the NCBI data base under accession code GSE217930, and are found here https://www.ncbi.nlm.nih.gov/geo/query/acc.cgi?acc=GSE217930
The processed single cell RNA seq data are available in the Zenodo data base, here "annotated_Sabah_data_21Oct2022.rds" https://doi.org/10.5281/zenodo.6973241 .

All other data generated in this study are provided in Supplementary Information/Source Data file.

Human Protein Atlas signatures for naive B cells can be found www.proteinatlass.org.

Research involving human participants, their data, or biological material

Policy information about studies with human participants or human data. See also policy information about sex, gender (identity/presentation), and sexual orientation and race, ethnicity and racism.

Reporting on sex and gender	Sex self assigned at hospital sites. Some, male sex bias, due to a high proportion of infected males across all malaria species, possibly because of infection risk of forest worker in recruitment areas (https://doi.org/10.1093/cid/ciy065). Written informed consent was obtained from all adult study participant or, in the case of children, parents or legal guardians.
Reporting on race, ethnicity, or other socially relevant groupings	NA
Population characteristics	Patients were between the ages of 3-60. Patients were treated with antimalarial treatment, age, self reported previous malaria, body temperateure, qPCR results, fever status, body temperature, days with fever, white blood cell count, haematocrit, anaemia liver palpabaility, spleen palpability and CMV status is presented in Supplementary Table S1.
Recruitment	Peripheral blood mononuclear cells (PBMCs) and plasma were obtained by convenance sampling of patients with uncomplicated malaria from previous conducted clinical trials and cohort studies of malaria conducted in Sabah Malaysia between 2010 and 2016. During this period, falciparum malaria was low in Sabah Malaysia, with an estimated incidence of 0.18/1000 people in 2011. These studies included a randomized controlled trial of artemether-lumefantrine against chloroquine, and a clinical efficacy study of oral artesunate followed by oral mefloquine, both conduced on patients with acute malaria at three district hospital sites (Kudat, Kota Marudu and Pitas), and a prospective observational study which malaria patients conducted at the tertiary referral center (Queen Elizabeth Hospital, Kota Kinabalu). Within these studies, non-pregnant patients (aged >3 years) with microscopy-diagnosed malaria who provided written informed consent were enrolled. For the drug treatment trials, treatment for individuals followed randomization. All individuals regardless of treatment were aparasitemic by 72 hours, confirmed by thick smear or PCR. For the observational study, treatment followed hospital guidelines: artemether-lumefantrine for falciparum and knowlesi malaria; chloroquine and primaquine or ACT for vivax malaria; and intravenous artesunate for severe malaria, or if deemed warranted by the treating clinician. Severe malaria was defined according to 2014 WHO guidelines, and patients with severe disease were not included in current immunology study. No treatment failures were reported. Across all trials/cohorts a higher proportion of infected males was observed across all malaria species, possibly because of infection risk of forest worker. Within these parent studies, blood samples were collected and archived for future studies from individuals at enrolment (acute malaria timepoint, day 0), and 7 and 28 days post-treatment. Additionally, samples were collected from individuals who had been living in the area in the preceding 3 weeks, who were negative for Plasmodium by microscopy, and who had no history of fever in previous 48 hours were as endemic healthy control samples (EC). Blood was collected in lithium-heparin collection tubes, then PBMCs and plasma were isolated from whole blood via density centrifugation with Ficoll-Paque prior to cryopreservation.<br><br>For the current immunology study, individuals for scRNAseq analysis were selected from parent studies based on sample availability of repeated blood samples at day 0, 7 and 28 after enrolment/treatment and confirmed uncomplicated falciparum mono-infection by PCR. For phenotypic analysis, further individuals from the same parent clinical trials/cohort were selected based on sample availability at day 0 (acute malaria and enrolment) with uncomplicated malaria and confirmed falciparum mono-infection by PCR. Not all day 0 samples also had a day 28 sample available, so additional day 28 (convalescence) time points were selected.
Ethics oversight	QIMR Berghofer Human Research Ethics Committee, Northern Territory Department of Health and Menzies School of Health

Research ethics committee, Medical Research and Ethics Committee, Ministry of Health, Malaysia

Note that full information on the approval of the study protocol must also be provided in the manuscript.

Field-specific reporting

Please select the one below that is the best fit for your research. If you are not sure, read the appropriate sections before making your selection.

[x] Life sciences [ ] Behavioural & social sciences [ ] Ecological, evolutionary & environmental sciences

For a reference copy of the document with all sections, see nature.com/documents/nr-reporting-summary-flat.pdf

Life sciences study design

All studies must disclose on these points even when the disclosure is negative.

Sample size	No sample size calculations were made. Individuals for scRNAseq analysis were selected from parent studies based on sample availability of repeated blood samples at day 0, 7 and 28 after enrolment/treatment and confirmed uncomplicated falciparum mono-infection by PCR. For phenotypic analysis, further individuals from the same parent clinical trials/cohort were selected based on sample availability at day 0 (acute malaria and enrolment) with uncomplicated malaria and confirmed falciparum mono-infection by PCR. Not all day 0 samples also had a day 28 sample available, so additional day 28 (convalescence) time points were selected.
Data exclusions	For scRNAseq all data that passed QC was included. For flow cytometry data, all data were included. For myeloid cytokine analysis, data was not included in analysis if <50 cell events were gated.
Replication	Major cell subset frequencies identified during single cell transcriptional analysis in each individual was confirmed by flow cytometry. Additional sequencing runs are not feasible.<br><br>For flow cytometry experiments, all panel optimization was performed on health malaria naive samples prior to analysis in this study. Panel optimization was check for reproducibility. For application to study samples, flow cytometry experiments were performed once due to limited cell numbers and patient samples
Randomization	Not relevant. Patients naturally acquired malaria and self-presented to local hospital. Malaria patients were treated according to hospital guidelines and participants recruited in prospective comparative study if consent was provided.
Blinding	Data generation for scRNAseq and flow cytometry analysis was done blinded to individual infection time points (day 0, 7, 28, healthy control) and patient characteristics (age, sex, parasitemia etc). These clinical data was only joined to flow cytometry and sequencing data after analysis/gating had been performed.<br><br>Blinding is not relevant in study recruitment as patients were presented to hospital with malaria for enrollment. This current study was not linked to parent study clinical trials

Reporting for specific materials, systems and methods

We require information from authors about some types of materials, experimental systems and methods used in many studies. Here, indicate whether each material, system or method listed is relevant to your study. If you are not sure if a list item applies to your research, read the appropriate section before selecting a response.

Materials & experimental systems

n/a	Involved in the study	
[ ]	[x]	Antibodies
[x]	[ ]	Eukaryotic cell lines
[x]	[ ]	Palaeontology and archaeology
[x]	[ ]	Animals and other organisms
[x]	[ ]	Clinical data
[x]	[ ]	Dual use research of concern
[x]	[ ]	Plants

Methods

n/a	Involved in the study	
[x]	[ ]	ChIP-seq
[ ]	[x]	Flow cytometry
[x]	[ ]	MRI-based neuroimaging

Antibodies

Antibodies used	Antibodies for ex vivo cell phenotyping comparison of scRNAseq samples:<br>-Antigen Fluorophore Clone Manufacturer Cat # Lot # -<br>CD16 BUV395 3G8 BD 563785 8339741<br>CD45RA BUV563 HI100 BD 565702 8060967

CD4 BUV737 SK3 BD 612748 9276448
CD27 BV421 M-T271 BD 562513 7086697
CD123 PacBlue 6H6 Biolegend 306043 B265977
CD56 BV510 HCD56 Biolegend 318340 B255646
CD127 BV570 A019D5 Biolegend 351307 B280140
CD11c BV650 Bu15 Biolegend 337238 B259943
CD8 BV711 RPA-T8 BD 563677 9129574
CD19 BV750 HIB19 Biolegend 302261 B270697
HLA-DR BV785 L243 Biolegend 307642 B283993
TCR γδ FITC B1 Biolegend 331208 B177066
CD3 AF532 UCHT1 Invitrogen 58-0038-42 2106888
CD14 PerCP-Cy5.5 M5E2 Biolegend 301824 B278177
ICOS PE DX29 BD 557802 8109815
CD86 PE-Daz594 IT2.2 Biolegend 305434 B267118
CXCR5 PE-Cy7 J252D4 Biolegend 356924 B244370
CD38 AF647 HIT2 Biolegend 303514 B271664
CD25 APC-R700 2A3 BD 565106 9150626
Vδ2 APC/Fire 750 B6 Biolegend 331420 B238651

Antibodies for ex vivo cytokine analysis:
-Antigen Fluorophore Clone Manufacturer Cat # Lot # -
CD19 BUV496 SJ25C1BD 612938 0233664
CD64 BUV737 10.1 BD 564425 1089969
CD14 BUV805 M5E2 BD 612902 1092492
CD86 BV480 2331 BD 566131 0163901
CD3 BB515 UCHT1 BD 564466 1050301
CD56 BV510 HCD56 Biolegend 318340 B318848
CD33 BV570 WM53 Biolegend 303417 B313863
CD123 BV605 6H6 Biolegend 306026 B265668
CD11c BV650 B915 Biolegend 117310 B323827
HLA-DR BV785 L243 Biolegend 307642 B307972
CCR2 PerCPCy5.5 K036C2 Biolegend 357204 B307717
Vδ2 APC B6 Biolegend 331418 B311264
CD16 AF700 3C78 Biolegend 302026 B333714
CD1c APC-FIRE L161 Biolegend 331545 B312469
LIVE/DEAD Fixable Blue Dead Cell Stain Invitrogen L23105 2176884
IFN-γ BUV395 B27 BD 563563 9287023
IL-6 BV421 C8-6 BD 501119 9127862
IL-4 BV711 MD4–25D2 BD 564112 0307148
TNF BV750 Mab11 BD 566359 1046844
IL-12 AF647 503-F7 Biolegend 565023 B268280
IL-10 PE-Dazzle JES–19F1 Biolegend 506812 B307282
MCP-1 PE-Cy7 5D3-F7 Biolegend 501818 B331376
IFN-α PE LT27-295 Miltenyi 130-092-602 5210409754
IL-1β FITC CRM56 Invitrogen 11-7018-42 2068718

Antibodies for NK/gd ex vivo cell phenotyping analysis:
-Antigen Fluorophore Clone Manufacturer Cat # Lot # -
CD27 AF700 M-T271 Becton-Dickinson 560611 0335723
VD1 APC TS8.2 Invitrogen 17-5679-42 2169733
VD2 APC-Fire B6 Biolegend 331420 B339574
CD19 BUV395 SJ25C1 Becton-Dickinson 563549 0230959
CD56 BUV563 NCAM16.2 Becton-Dickinson 612928 1139116
CD279 (PD1) BUV615 EH12.1 Becton-Dickinson 612991 0282760
CD16 BUV737 3G8 Becton-Dickinson 612787 0064610
CD3 BUV805 SK7Becton-Dickinson 612893 1043703
CD57 BV421 NK-1 Becton-Dickinson 563896 8352571
CD38 BV480 HIT2 Becton-Dickinson 566137 9219337
CD86 BV510 2331 (FUN-1) Becton-Dickinson 563461 0217361
CD14 BV570 M5E2 Biolegend 301831 B282108
CD366 (Tim-3) BV605 F38-2E2 Biolegend 345017 B288824
CD278 (ICOS) BV650 DX29Becton-Dickinson 563832 0212336
CD223 (LAG3) BV711 11C3C65 Biolegend 369319 B310749
HLA-DR BV750 L243 Biolegend 307672 B312755
IgD BV785 IA6-2 Biolegend 348242 B288812
CD98 FITC MEM-108 Biolegend 315603 B285093
HIF-1a PE 546-16 Biolegend 359703 B324004
Perforin PE-CF594 δG9 Becton-Dickinson 563763 0246012

CD21 PE-Cy7 B-ly4 Becton-Dickinson 561374 9337049
GranzymeB PerCP-Cy5.5 QA16A02 Biolegend 372212 B334337
LIVE/DEAD Fixable Blue Dead Cell Stain Invitrogen L23105 2176884

Antibodies for CD4 ex vivo cell phenotyping analysis
-Antigen Fluorophore Clone Manufacturer Cat # Lot # -
LAG3 BV421 Biolengend 369314 B340235
CD49b FITC Invitrogen 11-0498-42 2295556
CCR7 PerCP-Cy5.5 BD 561144 1193676
CD14 BUV395 BD 563561 1109248
CD19 BUV395 BD 563549 9259738
CD8 BUV496 BD 612942 0255680
CD3 BUV805 BD 612893 0177051
CD38 BV480 BD 566137 1193057
CD127 BV570 Biolengend 351308 B356494
CCR6 BV650 BD 563922 1210064
CXCR5 BV711 Biolengend 356934 B346460
TIM3 BV750 Biolengend 345056 B350567
CD4 BV785 Biolengend 317442 B313176
CD120b PE Biolengend 358404 B324783
CXCR3 PE-DAZ594 BD 562451 1109427
PD1 PE-CY7 BD 561272 1039451
ICOS APC-CY7 Biolengend 313530 B288985
CTLA4 BV605 Biolengend 369610 B336024
FOXP3 AF647 Biolengend 320114 B241845
LIVE/DEAD Fixable Blue Dead Cell Stain Invitrogen L23105 2176884

Validation

Antibody validations were performed by the suppliers per quality assurance documents provided by each supplier. All validation data are available on the manufacturer's websites.
Antibodies were purified by affinity chromatography and conjugated under optimal conditions by the manufacturer. Each antibody lot is FC quality control tested by immunofluorescent staining with flow cytometric analysis. Monoclonal antibodies have verified reactivity with human species and are for Research Use Only (RUO)

Flow Cytometry

Plots

Confirm that:

☒ The axis labels state the marker and fluorochrome used (e.g. CD4-FITC).

☒ The axis scales are clearly visible. Include numbers along axes only for bottom left plot of group (a 'group' is an analysis of identical markers).

☒ All plots are contour plots with outliers or pseudocolor plots.

☒ A numerical value for number of cells or percentage (with statistics) is provided.

Methodology

Sample preparation

Peripheral blood mononuclear cells (PBMCs) were obtained from patients with acute uncomplicated clinical Plasmodium falciparum malaria. PBMCs were isolated from whole blood via density centrifugation with Ficoll-Paque prior to cryopreservation.

Instrument

Cytek Aurora 5 laser instrument (UV-V-B-YG-R).

Software

Collected with SpectroFlo v3.0. Analysed with FlowJo v10.8.1.

Cell population abundance

No cell-sorting was performed in this study.

Gating strategy

Gating strategy for stains are exemplified in supplementary:

ex vivo cell phenotyping comparison of scRNAseq samples (Fig. S2)
- Lymphocytes identified by granularity and size with FSC-A v SSC-A, live cells were LIVE/DEAD BLUE-,CD14-, CD3+CD19- (T cells) CD3-CD19+ (B cells), CD3-CD19- (non T/B cells), then CD3+CD19-gamma-deltaTCR+ (gamma-delta T cell), CD3+CD19-γδTCR-CD4+CD8- (CD4 T cells), CD3+CD19-γδTCR-CD4-CD8+ (CD8 T cells) and CD3-CD19-CD56+ or CD3-CD19-CD56-CD16+ (NK cells).
- Myeloid cells identified by granularity and size with FSC-A v SSC-A, live cells were LIVE/DEAD BLUE-, CD19-, HLADR+, then CD14+CD16- (CD14 monocytes), CD16+ (CD16 monocytes), CD14-CD16- (dendritic cells), CD14-CD16-CD11c+CD123- (classical dendritic cells) and CD14-CD16-CD11c-CD123+ (plasmacytoid dendritic cells).

ex vivo cytokine analysis (Fig. S4A, S7A and S22A)
-Myeloid cells identified by granularity and size with FSC-A v SSC-A, live cells were LIVE/DEAD BLUE negative, CD3-CD19-, HLADR+CD56-, then CD14-CD1c+ (CD1c dendritic cells), CD14+CD1c- (CD14 monocytes), CD14-CD1c-CD123+CD11c- (plasmacytoid dendritic cells) and CD14-CD1c-CCDR2-CD33-plasmacytoiddendriticcell- (CD16 monocytes),
- NK cells were identified by granularity and size with FSC-A v SSC-A, live cells were LIVE/DEAD BLUE negative, CD3-CD19-, then CD56++CD16- (CD56++NK cells) and CD56dimCD16+ (Adaptive NK cells).
- B cells were identified by granularity and size with FSC-A v SSC-A, live cells were LIVE/DEAD BLUE negative, CD3-CD19+, CD56- (B cells)

ex vivo gdBNK cell phenotyping analysis (Fig. S7A, S11B and S20B).
- Doublets removed by FSC-A v FSC-H, lymphocytes identified by granularity and size with FSC-A v SSC-A, live cells were LIVE/DEAD BLUE-, CD14-, CD3+CD19- (T cells), CD3-CD19+ (B cells), CD3-CD19-CD56+ (NK cells), then CD3+CD19-Vδ2TCR+Vδ1TCR- (Vδ2+ γδ T cells), CD3+CD19-Vδ2TCR-Vδ1TCR+ (Vδ1+ γδ T cells), CD3-CD19+CD27highCD38high (Plasmablast), CD3-CD19+plasmablast-IgD+ (transitional/naive B cells), CD3-CD19+plasmablast-IgD+CD27+CD21+ (memory B cells), CD3-CD19+plasmablast-IgD+CD27+CD21- (activated memory B cells), CD3-CD19+plasmablast-IgD+CD27-CD21- (atypical B cells), CD3-CD19-CD56+PD1+ (PD1+ NK cells), CD19-CD56+PD1+CD56brightCD16- (CD56 bright NK cells) and CD19-CD56+PD1+CD56dimCD16+ (Adaptive NK cells).

ex vivo CD4 cell phenotyping analysis (Fig. S15A).
- Doublets removed by FSC-A v FSC-H, lymphocytes identified by granularity and size with FSC-A v SSC-A, live cells were LIVE/DEAD BLUE-CD14-CD19-, CD4+CD8-(CD4 T cells) then naive (CD45RA+), Tr1 (CD45RA-LAG3+CD49b+), Treg (CD45RA-Tr1-/FOXP3+CD127lo), Tfh (CD45RA-Tr1-/Treg-/CXCR5+), Tfreg (CD45RA-Tr1-/Treg+/CXCR5+), Th1 (CD45RA-Tr1-/Treg-/CXCR5-/CXCR3+CCR6-), Th2 (CD45RA-Tr1-/Treg-/CXCR5-/CXCR3-CCR6-) and Th17 (CD45RA-Tr1-/Treg-/CXCR5-/CXCR3-CCR6+).

☒ Tick this box to confirm that a figure exemplifying the gating strategy is provided in the Supplementary Information.
